# Effectiveness of Nonpharmacological Interventions in the Field of Ventilation: An Umbrella Review

**DOI:** 10.3390/ijerph20075239

**Published:** 2023-03-23

**Authors:** Neuza Reis, Luis Gaspar, Abel Paiva, Paula Sousa, Natália Machado

**Affiliations:** 1 Nursing Research, Innovation and Development Centre of Lisbon (CIDNUR), Rehabilitation Nurse, CHULC, 1900-160 Lisbon, Portugal; 2 RN Centro Hospitalar Universitário S. Joao, 4200-319 Porto, Portugal; 3 NursingOntos, Escola Superior de Enfermagem do Porto, 4200-072 Porto, Portugal

**Keywords:** health impact assessment, nonpharmacological interventions, review, nursing, rehabilitation

## Abstract

This umbrella review aimed to determine the effectiveness of nonpharmacological interventions in pulmonary ventilation and their impact on respiratory function. An individual with impaired ventilation displays visible variations manifested in their respiratory frequency, breathing rhythm ratio (I:E), thoracic symmetry, use of accessory muscles, dyspnea (feeling short of breath), oxygen saturation, diaphragm mobility, minute ventilation, peak flow, walking test, spirometry, Pimax/Pemax, diffusion, and respiratory muscle strength. Any variation in these markers demands the need for interventions in order to duly manage the signs and symptoms and to improve ventilation. Method: Systematic reviews of the literature published in English, Spanish, French, and Portuguese were used, which included studies in which nonpharmacological interventions were used as a response to impaired ventilation in adults in any given context of the clinical practice. The recommendations given by the Joanna Briggs Institute (JBI) for umbrella reviews were followed. This research took place in several databases such as MEDLINE, CINAHL Complete, CINHAL, MedicLatina, ERIC, Cochrane Reviews (Embase), and PubMed. The Joanna Briggs critical analysis verification list was used for the systematic review. The data extraction was performed independently by two investigators based on the data extraction tools of the Joanna Briggs Institute, and the data were presented in a summary table alongside the support text. Results: Forty-four systematic reviews, thirty randomized clinical essays, and fourteen observational studies were included in this review. The number of participants varied between n = 103 and n = 13,370. Fifteen systematic revisions evaluated the effect of isolated respiratory muscular training; six systematic revisions evaluated, in isolation, breathing control (relaxed breathing, pursed-lip breathing, and diaphragmatic breathing exercises) and thoracic expansion exercises; and one systematic review evaluated, in isolation, the positions that optimize ventilation. Nineteen systematic reviews with combined interventions that reinforced the role of education and capacitation while also aiming for their success were considered. The articles analyzed isolated interventions and presented their efficacy. The interventions based on respiratory exercises and respiratory muscular training were the most common, and one article mentioned the efficacy of positioning in the compromisation of ventilation. Combined interventions in which the educational component was included were found to be effective in improving pulmonary function, diffusion, oxygenation, and functional capacity. The outcomes used in each study were variable, leading to a more difficult analysis of the data. Conclusions: The interventions that were the focus of the review were duly mapped. The results suggest that nonpharmacological interventions used to optimize ventilation are effective, with a moderate to high level of evidence. There is a strong foundation for the use of the chosen interventions. The lack of studies on the intervention of “positioning to optimize ventilation” points out the need for a deeper analysis of its effects and for studies with a clear focus. This study supports the decisions and recommendations for the prescription of these interventions to patients with impaired ventilation.

## 1. Introduction

Respiratory function comprises ventilation, gas exchange, and cellular respiration [1]. Besides this division, it is central to consider respiratory function as a whole, as it is essential to maintain an adequate oxygen supply to the body [2].

Ventilation is the process of moving air into and out of the lungs with a certain frequency, respiratory rhythm, depth, and expiratory strength [3]. Pulmonary ventilation is crucial for air renewal in the lung areas in which gas exchange occurs [4].

Impaired ventilation leads to deep changes in the relationship between the affected person and the environment or people around said subject.

The applicability of an intervention to impaired ventilation depends on an adequate exam of the affected patient and on an analysis of all the data, which allows one to identify, relate, and duly hierarchize the issues that are targeted by the intervention.

Know-how on the patient, alongside the anatomy and physiology principles, is crucial for the prescription of an adequate therapeutical intervention.

The decision on the intervention goes far beyond the medical diagnosis; for instance, patients with the same diagnosis experience different signals, symptoms, and severities regarding their impaired ventilation. Additionally, the repercussions on the patient’s social participation and the limitations they experience are also very different and are individualized depending on exclusive environmental factors.

There are several systematic reviews on nonpharmacological interventions in individuals with impaired ventilation. Given that, this umbrella review is relevant to mapping the best available scientific evidence on the efficacy of said interventions, systematizing all the information on the patient’s evaluation and the implementation of an intervention in order to obtain optimal results. This mapping makes it possible to organize all the relevant information on this topic in which the data are evaluated, the data support the non-pharmacological intervention, and the results validate its effectiveness. It allows one to build a clinical rationale for nursing, increasing evidence-based practices, and contributing to improving the quality of care.

The preliminary research on the data banks JBI for Systematic Reviews and Implementation Reports, Cochrane, PROSPERO, PubMed, and CINAHL concluded that no umbrella reviews have been performed and that no progress has occurred for this subject. Therefore, this revision is relevant to discovering the best possible evidence; evaluating its quality; providing state-of-the-art knowledge on the effectiveness of the nonpharmacological interventions in the ventilatory domain, namely, the “training of the respiratory muscle”, “execution of techniques of breathing control”, “execution of positioning techniques”, and “execution of thoracic expansion techniques”; and mapping all the nursing interventions related to impaired ventilation. This mapping allows for the organization of all the relevant information on this subject to build a clinical rationale for nursing, increasing practices based on evidence, and contributing to an improvement in the quality of care.

## 2. Materials and Methods

Preliminary research on the data banks JBI for Systematic Reviews and Implementation Reports, Cochrane, PROSPERO, PubMed, and CINAHL concluded that no umbrella reviews have been performed and that no progress has occurred for this subject. Therefore, this revision is relevant to discovering the best possible evidence; evaluating its quality; providing the state-of-the-art knowledge on the effectiveness of the nursing interventions in the ventilatory domain, namely, “training of the respiratory muscle”, “execution of techniques of breathing control”, “execution of positioning techniques”, and “execution of thoracic expansion techniques”; and mapping all the nursing interventions related to impaired ventilation. 

### 2.1. Review Questions

What data allow the evaluation of patients with ventilatory compromisation and support nonpharmacological interventions?

What is the efficacy of the nonpharmacological interventions in the ventilatory domain, namely, “training of the respiratory muscle”, “execution of techniques of breathing control”, “execution of positioning techniques”, and “execution of thoracic expansion techniques”?

### 2.2. Inclusion Criteria

Participants: Patients with impaired ventilation regardless of their medical diagnosis [5] who were adults 18 years old or over were included.

Intervention: Systematic reviews that evaluate the effectiveness of nonpharmacological interventions on adults were included—nonpharmacological interventions on the ventilatory domain, namely, “training of the respiratory muscle”, “execution of techniques of breathing control”, “execution of positioning techniques”, and “execution of thoracic expansion techniques”—without limitations on frequency/intensity, the intervention prescriber, or the intervention executioner, and they were isolated or combined. We included studies in which nonpharmacological interventions were used as a response to impaired ventilation in adults in any given context of the clinical practice.

Comparator: This umbrella included systematic reviews that compared an intervention with placebo or other nonpharmacological interventions.

Outcomes: The following were considered to be primary outcomes: respiratory rate, respiratory flow, symmetry of the respiratory movement, dyspnea, diaphragm mobility, tidal volume, oxygen saturation, pulmonary function (spirometry), diffusion, peak flow, Pimax and Pemax. The impact on the patient’s functional capacity (walking test) was evaluated as a secondary outcome. The majority of the systematic reviews did not relate the symmetry of respiratory movement, focusing on respiratory frequency, pulmonary function, and functional capacity.

Context: There were no limitations of context in this umbrella review.

Types of studies: Quantitative systematic reviews (with or without meta-analyses), systematic reviews of mixed methods, studies with pre- and postintervention comparisons (with and without interventions), and randomized studies were included. All the studies were focused on the evaluation of the efficacy of interventions, focusing on impaired ventilation.

Articles in English, French, Spanish and Portuguese, with no temporal limitations, were considered. Nonpharmacological and nonsurgical interventions targeting ventilation optimization in children were included. SNOMED_CT was used to classify interventions [6]. 

This review was done according to the Joanna Briggs Institute (JBI) Reviewer’s Manual [7] and considering the guidelines of Preferred Reporting Items for Systematic Reviews and Meta-Analyses Extension for Scoping Reviews (PRISMA-ScR) Checklist [8,9].

The protocol was registered in the OSF on 2021-06-17 with identification number DOI 10.17605/OSF.IO/NGFQC.

### 2.3. Search Strategy

The searching of databases occurred in June 2021. A comprehensive review, without temporal limitations, was performed in order to identify all the relevant search syntheses [7].

The following electronic databases were consulted: Scopus—481 results; Web of Science—406 results; Joanna Briggs Institute Database of Systematic Reviews and Implementation Reports—0 results; CINAHL Complete (EBSCO), CINAHL Plus with Full Text, MedicLatina, MEDLINE with Full Text, MEDLINE, and ERIC—202 results; and Cochrane Database of Systematic Reviews, Database of Abstracts of Reviews of Effects, PubMed, and Embase—9 results. The keywords are described in Supplementary S1 (database search terms). An additional search was done in the PEDro database—56 results, with the keywords “respiratory therapy”, “impaired ventilation”, and “systematic review”.

### 2.4. Study Selection

**Figure 1:**
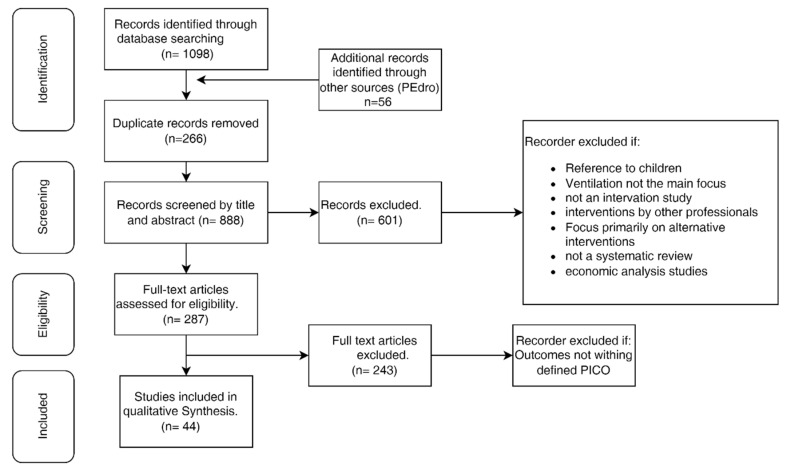
Flowchart of the study selection and inclusion process.

After researching every database, all of the articles were included in EndNote VX7 (Clarivate Analytics, PA, EUA), and duplicates were excluded. Two revisors (RN and GL) examined, independently, all of the titles and abstracts of the articles, and, upon any divergence, the convergent points were taken into consideration. All divergencies were discussed until consensus. According to the inclusion criteria, the studies eligible for full reading were evaluated in detail. The results of the research were presented in a flowchart (Figure 1). After a descriptive evaluation of each study, the data were extracted to a data sheet and were developed according to the goals and investigation questions as per the Joanna Briggs Institute methodology for umbrella reviews [7]. 

Assessment of methodological quality:

According to the inclusion criteria, the studies eligible for full reading were evaluated by two independent investigators (RN and GL) in order to validate their methodological quality before being included in the umbrella review. For this purpose, JBI Critical Appraisal Checklist for Systematic Reviews and Research Syntheses was used as the evaluation instrument. Any disagreement between the investigators was solved via discussion or with the support of a third investigator (SP). The results were presented in a narrative shape. To evaluate the methodological quality, the following scale was used: a total of 0–3 was considered a very low-quality score; 4–6 was considered a low-quality score; 7–9 was considered a moderate-quality score; and 10–11 was considered a high-quality score. Studies with a minimum score of 4 were included in the umbrella review.

### 2.5. Data Extraction

After a descriptive evaluation of each study, the data were extracted by two independent investigators (RN and GL) to a data sheet and were developed according to the Joanna Briggs Institute methodology for umbrella reviews [7]. 

The extracted data (author, type of study, year and place of publishment, population, data, type of intervention, and results) were summarized on a table to allow for analysis and for conclusions to be made.

### 2.6. Data Synthesis

The categories of results were the basis for the analysis of the systematic reviews. Analysis charts were made and a narrative synthesis was performed to meet the goal of the umbrella review alongside a clear description of the interventions. The presented charts included data on the number of studies, total number of participants, and interventions used to tackle impaired ventilation. The overlapping of primary studies included in the systematic reviews was duly verified to avoid duplicates.

The results of the umbrella review were provided in an ‘‘Evidence Summary’’ including systematic reviews; interventions; and a visual indicator of the intervention efficacy using the three traffic light colors, green for effective, orange for slightly effective in comparison with the control, and red for noneffective or less effective than the control [7]. 

GRADE (Grading of Recommendations, Assessment, Development and Evaluation) was used to evaluate the quality of the evidence [10]. This scale has 4 levels: high, moderate, low, and very low. The quality of the evidence was classified according to the risk of bias, inconsistency, imprecision, and bias. The metrics used for systematic reviews, such as standard deviation, weighted mean difference, and Hedges’ effect, were taken into consideration.

## 3. Results

### 3.1. Review Selection

After the sorting of 1154 articles, 287 were selected for full reading, and 44 were included due to meeting the defined criteria. The selection process is described in Figure 1.

### 3.2. Methodological Quality

The methodological quality was evaluated as per the JBI criteria (Table 1).

The two investigators agreed on the selected studies, hence the noninclusion of the third investigator. 

As per the JBI criteria for systematic reviews, research, and synthesis [7], 30 high-quality and 14 moderate-quality systematic reviews were included. The number of met criteria ranged between 8 (minimum) and 11 (maximum).

Twenty-nine systematic reviews evaluated the bias probability. None were excluded due a lack of methodological quality.

The methodological evaluation of the systematic reviews based on the JBI evidence levels followed [7]. The reviews were catalogued as level 1, with 30 being in level 1A (systematic review of randomized controlled trials) and 26 in level 1B (systematic review of RCTs and other study designs).

A total of 18 of the selected reviews were randomized controlled trials (RCTs), and 24 comprised meta-analyses. The articles regarding respiratory muscle training were the ones in which meta-analyses were conducted, and they presented the results via a narrative synthesis (Supplementary S2).

Supplementary S2 summarizes the features of the 44 studies, and they were classified as per their study design, population, place and year of publishing, interventions, data and results on the interventions, general quality classification, and data relevance. 

All studies were published between 2005 and 2021 and were from four continents—Oceania (Australia (five) and New Zealand (one)), Asia ((Israel (one), China (five), Malaysia (one), Korea (one), and India (one)), America (the USA (two), Canada (four), and Brazil (seven)), and Europe (the UK (three), Ireland (two), the Netherlands (one), Spain (three), Portugal (two), Germany (one), Belgium (one), Cyprus (one), Denmark (one), and France (one)).

The number of participants ranged between n = 113 and n = 13,370, and they were aged > 18 years. One of the studies had a population aged > 16 [38]. Twelve systematic reviews were carried out on a population with DPOC [13,15,16,21,30,35,40,42,43,51,53,54]. Two systematic reviews were carried out on a population with asthma [47,49], two were carried out on a population with amyotrophic lateral sclerosis [19,34], two were carried out on a population with multiple sclerosis [44,55], two were carried out on a population with Parkinson’s disease [11,20], three were carried out on people who were poststroke [12,18,37], one was carried out on a population with pulmonary fibrosis [25], and three were carried out on a population with lung cancer [14,26,45]. Six reviews focused on preoperatory preparation [24] and postoperatory preparation [31,33,52,56], and three reviews focused on the respiratory exercises for abdominal surgeries [17,50,57]. Four studies pointed out the efficacy of the interventions on patients with cardiac failure [23,39,48,58], and one pointed the significance of the interventions on patients with a Fontan circulation [46]. One study related positioning to lung function [27].

Forty-four systematic reviews were included in the final analysis, meeting the central objective—to investigate the evidence for interventions in patients with impaired ventilation. The interventions that were in the scope of the study were present in all the systematic reviews.

### 3.3. Intervention Characteristics (Type/Frequency/Duration/Intensity)

There was a great variety of respiratory exercises, but the most used exercises were diaphragmatic breathing and brake-labial breathing. No pattern regarding frequency, durability, and prescribed exercises was found, with only one study describing the frequency and durability of each respiratory exercise [49]. 

Eleven studies presented a program consisting of various interventions, such as thoracic expansion exercises, respiratory control, relaxation exercises, inspiratory muscle training, and the use of an incentive spirometer [14,15,16,21,22,25,33,34,41,42,43].

An article referred the efficacy of the rehabilitation program to the inclusion of lung volume recruitment training [34]. The therapeutical protocols varied between 6 and 8 weeks with two to three sessions per week (each between 60 and 90 min).

Five studies were focused on the significance of the respiratory control exercises (pursed-lip breathing, diaphragmatic breathing exercises, and relaxed breathing) [13,26,49,52,57]. Two studies specified the importance of the use of an incentive spirometer during the respiratory exercises [26,49]. Four studies displayed inspiratory muscle training (IMT) as an effective intervention against impaired ventilation [11,20,26,50].

As for the intervention of inspiratory muscle training (IMT), thirty studies showed its effectiveness in the face of compromised ventilation. One study pointed out that combined exercises (inspiratory muscle training and expiratory muscle training) presented better outcomes than isolated inspiratory muscle training [40]. 

There were three types of inspiratory muscle training used in the program: normocapnic hyperpnea, inspiratory resistance training, and threshold load. These exercises, when associated with pulmonary expansion, were effective on individuals with fluctuations in their pulmonary function [37,56]. Four studies focused on the significance of inspiratory muscle training for patients in critical stages [22,29,32,36], reinforcing its role in ventilatory withdrawal [22,29,32,36]. This intervention was well tolerated by patients in critical stages, allowing for a decrease in the time of invasive ventilation, the duration of noninvasive ventilatory support, and the time spent in intensive care units [29,32,36]. 

Inspiratory muscle training can be considered to be an adjuvant intervention, mainly for those who do not stick to conventional rehabilitation and who present respiratory muscular weaknesses [23]. Inspiratory muscular training, performed for 6 weeks as a complementary intervention in a program for neurological rehabilitation, allowed for an improvement in respiratory capacity and was associated with an increase in the pulmonary volume and exercise capacity. Improvements in dyspnea and quality of life (evaluated with the Medical Outcomes Study Short-Form 12-item Questionnaire) were also verified [37].

The prescription of the intervention of “respiratory muscle training” (Table 2) has to take the following into account: weight (intensity), frequency, durability, endurance, and the results attained after the interruption of training [12,36,39,48,53,55,56,57]. 

The training protocols in which expirations were done in a certain way had better results for Pemax, and the essays focused on respiratory muscle training (inspiratory) had better results for Pimax [44]. One study defined a high intensity program, and, when the intervention was performed, the intensity was 50% of Pimax [23]. The training protocol lasted for 6 to 8 weeks on average with a frequency of three to seven times per week one to three times per day for approximately 30 min per session. All the studies used an increasing method, starting with a weight of 30 to 50% of the Pimax until 60 to 70% was reached. The training weight adjustment was calculated according to the results obtained during the program. The maximum inspiratory pressure (Pimax), walking distance, and dyspnea improved in all the studies, mainly in those with a weight of 60% of the Pimax and with training six times a week for 12 weeks. In patients with chronic heart failure, IMT allowed an increase in muscular strength, walking distance, and dyspnea, especially when training with 60% of Pimax six times a week for 12 weeks [23]. 

In view of the interventions regarding thoracic expansion, one study considered the modalities of expiratory resistance (CPAP, EPAP, BiPAP, and VNI) as postoperatory respiratory interventions. Ventilation with high expiratory resistance (CPAP, EPAP, BiPAP, and VNI) decreased the risk of pulmonary complications [39]. Three studies included CPAP (continuous positive airway pressure) as a supporting technique in thoracic expansion with benefits in ventilation [19,21,33].

One study evaluated the positioning of the patient to optimize ventilation and its effect on pulmonary function considering that physiology and pulmonary functions are influenced by body position [27].

**Figure 2:**
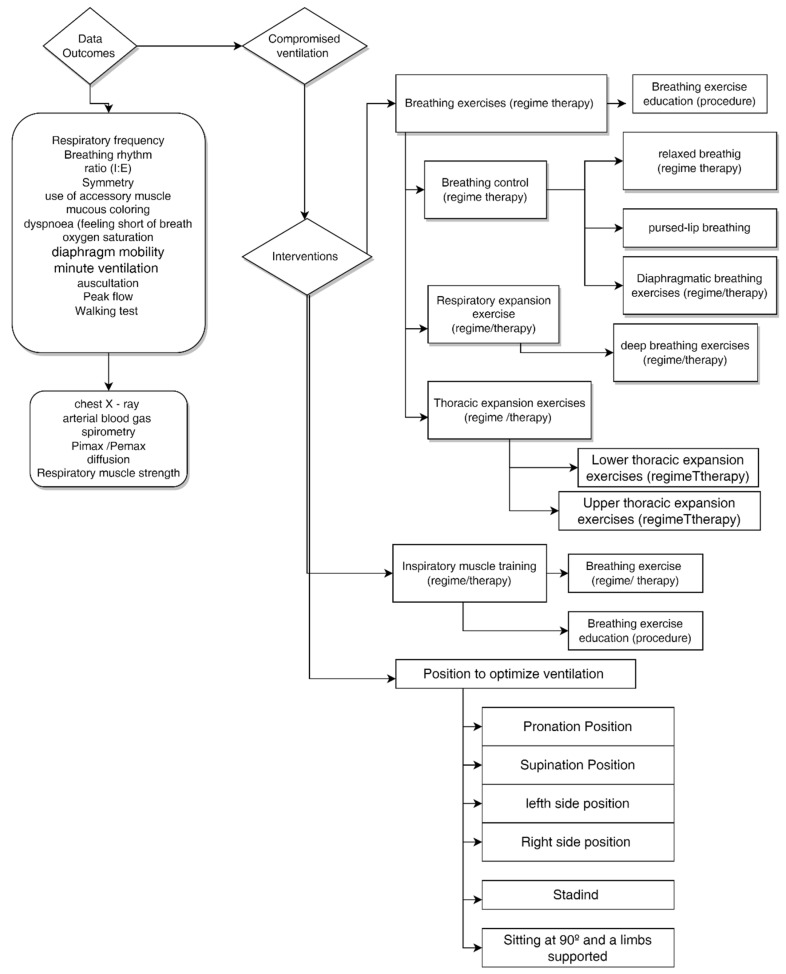
Mapping of interventions and their relationship with data/outcomes.

The education, know-how, and capacitation of the patients were also described in the studies, with their roles being highlighted in five of them [15,16,21,24,41]. This study allowed the mapping of the interventions and their relationship with the data/outcomes, which supports the applicability of the interventions, as shown in Figure 2.

### 3.4. Review Findings—Effects of Compromised Ventilation: Spirometry, Pulmonary Function, and Pimax/Pemax

All the studies pointed to an improvement in pulmonary function (FVC, FEV1, FEV/FVC, spirometry, Pimax, and Pemax) upon performing respiratory control and thoracic expansion exercises [13,26,49,50,52].

Respiratory control exercises, when associated with thoracic expansion exercises and an incentive spirometer, can improve pulmonary function, maximum inspiratory pressure (Pimax), and maximum expiratory pressure (Pemax).

In addition, these exercises can also decrease postoperatory pulmonary complications from pulmonary surgeries and decrease the incidence of pneumonia and atelectasis [26].

Respiratory exercises (respiratory control, pulmonary expansion, and incentive spirometry) presented medium- and long-term benefits in regard to respiratory muscle strength [57]. There was no clear evidence on the benefits of respiratory control exercises (pursed-lip breathing, diaphragmatic breathing exercises, and relaxed breathing) with respect to long-term ventilation when performed in isolation [52].

Respiratory muscle training (inspiratory and expiratory muscle training) and incentive spirometry improves respiratory muscle strength, peak expiratory flow (PEF), and pulmonary function [11,18,25,28,37,38,40,44,46,48,58].

Concerning the Parkinson’s disease patients, inspiratory muscle training (IMT) responded to their functional changes, correcting ventilation asynergies [20]. Moreover, IMT presented consistent improvements in functional capacity when performed in the preoperatory stage of abdominal, thoracic, and cardiac surgeries, allowing early weaning after operation [24,33,38,41,56].

Inspiratory muscle training (normocapnic hyperpnea, inspiratory resistance training, and threshold load) was an effective intervention for improving pulmonary function and cardiopulmonary resistance and decreasing the incidence of pulmonary infections in poststroke patients [18,37]. 

The intervention of “positioning to optimize ventilation” was effective with great improvements in pulmonary function considering that physiology and pulmonary functions are influenced by body position [27].

### 3.5. Frequency and Breathing Rate, Tidal Volume, Oxygen Saturation, and Dyspnea

Respiratory control exercises, when associated with pulmonary expansion exercises and an incentive spirometer, decrease one’s breathing rate and hyperventilation, allowing for better control of the diaphragm’s movements; increase tidal volume and oxygen saturation; and improve breathing difficulties and shortness of breath/dyspnea [49,52].

Respiratory muscle training (inspiratory and expiratory muscle training e incentive spirometry) improves shortness of breath/dyspnea, which was evaluated using the Borg scale [11,18,25,28,37,38,40,44,46,48,58]. 

Inspiratory muscle training (IMT) improves shortness of breath/dyspnea and quality of life [18,23,44,46,48,51,53,54,58].

### 3.6. Functional Capacity

Respiratory control exercises, in association with thoracic expansion exercises, improve functional capacity, which was evaluated through a 6 MW [13,26] and 12 MWT [52]. 

Inspiratory muscle training (IMT) presented visible benefits in regard to the levels of physical activity and functional capacity (6 MWT) (12 MWT) and improved shortness of breath/dyspnea and quality of life [18,23,44,46,48,51,53,54,58]. Peak oxygen consumption increased significantly post-training [18,46]. An improvement in peak VO2 was verified [23,24,48,58,59].

Table 3 presents a summary of the most frequent data evaluated in the systematic reviews.

Table 4 presents evidence on the interventions exhibited in the systematic reviews included in the study.

## 4. Discussion

This review identified a moderate to high quality for the majority of the interventions—Table 4 and Table 5.

The cause and effect were verified in all the revisions (pathology → structural deficiencies → functional limitations → quality of life) to be related to severity, extension and/or deficit reversibility, age, sex, lifestyle, and intervention.

The best evidence suggests that the studied interventions, respiratory control exercises, thoracic expansion techniques, respiratory muscle training, and positioning, have benefits in pulmonary function, functional capacity, dyspnea, and health-related quality of life [14,15,16,25]. 

Upon impaired ventilation, respiratory control exercises (relaxation and abdominal diaphragmatic technique) and thoracic expansion have the purpose of decreasing the respiratory workload, improving alveolar ventilation, and improving the coordination and efficiency of the respiratory muscles. These interventions contribute to ventilatory supply and distribution, a decrease in energy consumption, and a decrease in the use of the accessory muscles [1].

Directed ventilation consists in diaphragmatic ventilation with a great tidal volume and low frequency. The studies point to an improvement in alveolar ventilation and oxygen saturation and the consequent normalization of the resting respiratory rate. Usually, the same is associated with the relaxation techniques of the upper thorax muscles, shoulders, and upper limbs. Literacy (education and the patient’s capacitation towards good practices in the execution of the respiratory control techniques) is crucial to the success and efficacy of the performed interventions [2,4].

Breathing exercises and education on respiratory control techniques and pulmonary expansion exercises can improve pulmonary function (increase in VEF1, CVF, and VEF1/CVF), impacting one’s breathing pattern and functional evaluation and presenting improvements in the six-minute walk test (6 MWT).

The correct ventilatory pattern is the one that allows efficient ventilation with the lowest energy expenditure. In cases of impaired ventilation, the ventilatory pattern suffers changes, as well as the location, frequency, rhythm, depth, and inspiration/expiration ratio, as an involuntary response to optimize the oxygen supply.

Changes in the ventilatory pattern imply major oxygen consumption, a large workload, a bigger energy expenditure, and dyspnea aggravation. Ventilatory asynchrony, regardless of the cause, is minimized through adequate interventions such as education on respiratory control, breathing rate control, tidal volume, abdominal movement control, and improvements in peripheral saturation.

Most of the included studies did not apply the interventions (respiratory exercises) in isolation but as an integral part of an intervention program composed of respiratory muscle training, positioning, and education on the disease. Their importance as primary interventions in patients with compromised ventilation is evident. There is some evidence that the structured use of respiratory exercises with respiratory muscle training in the pre- and postoperatory stages can improve postoperatory recovery.

Preoperatory interventions significatively decrease the risk of the development of pulmonary complications in the postoperatory stage and reduce the hospital length of stay [29].

According to the results, the use of an incentive spirometer, allowing a maximum sustained inspiration with visual feedback on the inhaled volume, is effective in the prevention of pulmonary complications (atelectasis and respiratory infections) after high abdominal and thoracic surgeries. Its applicability is conditioned by the targeted pulmonary region in regard to ventilation and lung volume. An abdominal or costal ventilatory pattern can be chosen and can be optimized with adequate positioning [60,61].

The ventilation pump is responsible for keeping an adequate level of alveolar ventilation, with the respiratory muscles playing a big part. The inability of the inspiratory muscles to generate adequate strength can lead to the failure of the ventilation pump, leading to hypoventilation and global respiratory failure.

The prescription of respiratory muscle training is dependent on the correct identification of the root cause of muscular weakness. The studies point to its efficacy in impaired ventilation. The specific training of the respiratory muscles is connected to an improvement in pulmonary function, dyspnea, and cough and the prevention of pulmonary infections. This data is in accordance with the data from the scientific literature [1,62]. Load weight, frequency, duration, and specificity should be considered upon delineation of the respiratory muscle training program. According to the studies, the most used training programs were voluntary isocapnia and resistance breathing. These interventions can be considered given their moderate to high evidence, but their applicability should be considered case by case upon evaluation.

The interventions focused on thoracic expansion with a device, and the expiratory resistance modalities (CPAP, EPAP, BiPAP, VNI) presented low to moderate evidence.

Only one article was focused on positioning on its own, and the others referred to the need for adequate positioning to maximize ventilation. The best evidence suggests that positioning is a great strategy to be implemented in patients with impaired ventilation when regional alveolar ventilation and global changes in ventilation/perfusion (hypoxemia) are detected [63]. Therapeutic positioning is the primary intervention used to promote arterial oxygenation due to its immediate effect and direct oxygen supply [2]. As per Hough (2001), positioning is the main intervention for an unstable patient in intensive care, reestablishing ventilation and avoiding the interference of abdominal pressure in the pulmonary volume [61].The effect of positioning in the respiratory system is connected to the gravitational stimulus—the shift of the decubitus to the bipedal position allows an increase in the total pulmonary capacity, an increase in the residual functional capacity, an increase in tidal volume and pulmonary compliance, an increase in PaO2 and diaphragmatic excursion, a decrease in the respiratory workload, and an increase in secretion mobilization. Surveillance and monitoring are crucial to the results [2].

Factors such as age, physiology, respiratory pathology, concurrent diseases (cardiac, neurological, vascular, metabolic, or oncological diseases), the extension and severity of the respiratory dysfunction, obesity, thoracic/abdominal pain, and the need for mechanic ventilation can condition the response to a lengthy positioning. Therefore, prescribing adequate positioning to optimize the oxygen supply and to decrease the respiratory workload and dyspnea through an increase in the respiratory muscles’ efficacy is dependent on the patient’s response, and monitoring is crucial to evaluating the efficiency of the intervention [4].

The repercussions of the changes in/limitations of the activity are very individualized since the patient’s adaptation to the intervention is related not only to the pathology but also to their environmental and personal context, making education and capacitation imperative to improving the patient’s health, which is a highlight of the present review [15,16,21,24,29].

### Limitations

This umbrella review investigated the efficacy of interventions targeted at patients with impaired ventilation. All the included reviews are methodologically robust as is intended in umbrella reviews [7]. The evidence on the interventions was defined as per the evaluation of the results obtained in the primary studies included in the systematic reviews.

In some studies, the interventions were not clearly defined in regard to frequency, duration, and intensity, limiting their degree of recommendation. A more robust definition of the interventions would allow for a most consistent analysis.

Few studies evaluated the interventions in isolation, which compromised their comparison and analysis. 

Articles that evaluated the outcomes of patients with thoracic pump failure and other pathological conditions were not isolated, as it was not possible to isolate the nonpharmacological interventions and to present their results, which would be a big elimination. This is suggested as a priority for future research studies.

The systematic description of the interventions’ contraindications, which were not covered in the included reviews, would better clarify their limitations, reinforcing their applicability.

## 5. Conclusions

The data and intervention mapping allowed for the organization of the information relevant to setting up critical thinking, refining evidence-based practices, and improving the quality of life of patients with impaired ventilation.

Interventions consisting of respiratory control exercises, thoracic expansion, respiratory muscle training, and position techniques for breathing improvement are efficient in patients with impaired ventilation. The best possible evidence in this umbrella review points out their efficacy, and their application is recommended.

Respiratory control exercises, in association with thoracic expansion and an incentive spirometer, are a great strategy for impaired ventilation. They improve pulmonary function and medium- to long-term Pimax and Pemax. In addition, they allow for a decrease in respiratory frequency, better control of hyperventilation and diaphragm movement, an improvement in tidal volume, a raise in oxygen saturation, an improvement in respiratory difficulties and feelings of shortness of breath/dyspnea, and an improvement in functional capacity (GRADE A: JBI Grades of Recommendation) [63,64].

Breathing control exercises (pursed-lip breathing, diaphragmatic breathing exercises, and relaxed breathing) are efficient in long-term ventilation (GRADE B: JBI Grades of Recommendation) [63,64].

Inspiratory muscle training is an effective intervention for impaired ventilation, improving the feelings of shortness of breath/dyspnea, respiratory muscle strength, and pulmonary function and presenting concrete benefits in oxygenation, gas exchange, cardiac function, and functional capacity (GRADE A: JBI Grades of Recommendation) [63]. This intervention also presents a GRADE A recommendation for critical patients, decreasing the time of invasive ventilation.

Positioning to optimize ventilation is an effective strategy in patients with impaired ventilation (GRADE B: JBI Grades of Recommendation) [63,64].

Most of the reservations regarding the efficacy of said interventions can be linked to the fact that most of the studies were not focused on isolated interventions.

Nevertheless, high-quality systematic reviews and a strong theoretical basis led to the application and prescription of these interventions.

Future evidence must be generated and synthesized in the areas identified as having a knowledge gap, that is, the intervention of positioning to optimize ventilation, so that the intervention can be carried out with greater consistency, ascertaining its advantages.

In addition, systematic reviews should clearly adopt an isolated intervention and instituted program as well as analyze the contraindications of each intervention.

## Figures and Tables

**Table 1:** Critical appraisal results of eligible systematic reviews.

Citation	Q1	Q2	Q3	Q4	Q5	Q6	Q7	Q8	Q9	Q10	Q11	Quality
McMahon, L., Blake, C., & Lennon, O. (2021) [11]	Y	Y	Y	Y	Y	Y	Y	Y	N	Y	Y	High
Wu F, Liu Y, Ye G, Zhang Y (2020) [12]	Y	Y	Y	Y	Y	Y	Y	Y	Y	Y	Y	High
Ying Yang, Liuyi Wei, Shizhen Wang, et al. (2020) [13]	Y	Y	Y	Y	Y	Y	Y	Y	N	Y	Y	High
Chorattas A; Papastavrou, E; Charalambous A; Kouta C (2020) [14]	Y	Y	Y	Y	Y	Y	U	Y	Y	Y	Y	High
Habib GMM, Rabinovich R, Divgi K, et al. (2020) [15]	N	Y	Y	Y	U	Y	Y	N	N	Y	Y	Moderate
Michael Hindelang, Florian Kirsch & Reiner Leidl (2020) [16]	Y	Y	Y	Y	N	Y	Y	N	Y	Y	Y	High
Kokotovic, D.; Berkfors, A.; Gögenur, I.; et al. (2020) [17]	U	Y	Y	Y	Y	Y	Y	Y	Y	Y	Y	High
Zhang, X.; Zheng, Y.; Dang, Y.; et al (2020) [18]	Y	Y	Y	Y	Y	Y	Y	Y	Y	Y	Y	High
Rosa Silva JP, Santiago Júnior JB, Dos Santos, et al. (2020) [19]	Y	Y	Y	Y	Y	Y	N	N	N	Y	Y	Moderate
van de Wetering-van Dongen, V. A., Kalf, J. G., van der Wees, P. J., Bloem, B. R., & Nijkrake, M. J. (2020) [20]	N	Y	Y	Y	Y	Y	Y	Y	Y	Y	Y	High
Machado A, Matos Silva P, Afreixo V, Caneiras C, Burtin C, Marques A.(2020) [21]	Y	Y	Y	Y	Y	Y	Y	Y	Y	Y	Y	High
Desjardins, M., & Bonilha, H. S. (2020) [22]	Y	Y	N	Y	Y	Y	N	N	U	Y	Y	Moderate
Azambuja, A., de Oliveira, L. Z., & Sbruzzi, G. (2020) [23]	Y	Y	Y	Y	Y	Y	Y	Y	Y	Y	Y	High
Bolger JC, Loughney L, Tully R, et al. (2019) [24]	Y	Y	Y	Y	Y	Y	N	N	N	U	Y	Moderate
Yu X, Li X, Wang L, et al. (2019) [25]	Y	Y	Y	Y	Y	Y	Y	Y	Y	Y	Y	High
Wang YQ, Liu X, Jia Y, Xie J. (2018). [26]	Y	Y	Y	Y	Y	Y	Y	Y	Y	Y	Y	High
Katz, S., Arish, N., Rokach, A., Zaltzman, Y., & Marcus, E. L. (2018) [27]	U	Y	Y	Y	Y	Y	Y	Y	Y	Y	Y	High
Sadek Z, Salami A, Joumaa WH, et al. (2018) [23]	Y	Y	Y	Y	Y	Y	Y	Y	Y	Y	Y	High
Lee, E.N.;Kim M.J. (2019) [28]	Y	Y	Y	Y	Y	Y	Y	Y	Y	Y	Y	High
Vorona S, Sabatini U, Al-Maqbali S, et al. (2018) [29]	U	Y	Y	Y	Y	Y	Y	Y	Y	Y	Y	High
Torres-Sánchez I, Cruz-Ramírez R, Cabrera-Martos I, et al (2017) [30]	Y	Y	N	Y	Y	Y	N	N	N	Y	Y	Moderate
Narayanan AL, Hamid SR, Supriyanto E. (2016) [31]	Y	Y	Y	Y	Y	Y	Y	Y	Y	Y	Y	High
Volpe MS, Aleixo AA, Negreiros de Almeida PR (2016) [32]	Y	Y	Y	Y	Y	Y	Y	Y	U	Y	Y	High
Gomes Neto M, Martinez BP, Reis HF, Carvalho VO. (2016) [33]	Y	Y	Y	Y	Y	Y	Y	Y	U	Y	Y	High
Macpherson CE, Bassile CC (2016) [34]	Y	Y	Y	Y	Y	Y	Y	Y	Y	Y	Y	High
Mohammed J, Da Silva H, Van Oosterwijck J, Calders P. (2016) [35]	N	Y	Y	Y	Y	Y	N		U	Y	Y	Moderate
Elkins M, Dentice R. (2015) [36]	Y	Y	Y	Y	Y	Y	Y	N	Y	Y	Y	High
Martín-Valero R, De La Casa Almeida M, Casuso-Holgado MJ, Heredia-Madrazo A. (2015) [37].	Y	Y	Y	Y	Y	Y	Y	Y	U	Y	Y	High
Mans, C. M., Reeve, J. C., & Elkins, M. R. (2015) [38]	Y	Y	Y	Y	Y	U	Y	Y	U	U	Y	Moderate
Montemezzo D, Fregonezi GA, Pereira DA, Britto RR, Reid WD. (2014) [39]	Y	Y	Y	Y	Y	Y	Y	Y	Y	Y	Y	High
Neves LF, Reis MH, Plentz RD, Matte DL, Coronel CC, Sbruzzi G. (2014) [40]	Y	Y	Y	Y	Y	Y	Y	Y	N	Y	Y	High
Snowdon D, Haines TP, Skinner EH. (2014) [41]	Y	Y	Y	Y	Y	Y	Y	Y	N	N	Y	Moderate
Osterling K, MacFadyen K, Gilbert R, Dechman G. (2014) [42]	Y	Y	N	Y	Y	Y	N	U	Y	Y	Y	Moderate
Jácome, C; Marques, A. (2014) [43]	Y	Y	Y	Y	Y	Y	Y	Y	N	Y	Y	High
Martín-Valero, R., Zamora-Pascual, N., & Armenta-Peinado, J. A. (2014) [44]	Y	Y	Y	Y	Y	Y	Y	N	N	Y	Y	Moderate
Singh F, Newton RU, Galvão DA, Spry N, Baker MK. (2013) [45]	U	Y	Y	Y	Y	Y	U	N	N	Y	Y	Moderate
O’Doherty AF, West M, Jack S, Grocott MP. (2013) [46]	Y	Y	Y	U	Y	Y	N	N	N	Y	Y	Moderate
Silva IS, Fregonezi GA, Dias FA, Ribeiro CT, Guerra RO, Ferreira GM. (2013) [47]	Y	Y	Y	Y	Y	Y	Y	Y	Y	Y	Y	High
Smart NA, Giallauria F, Dieberg G. (2013) [48]	Y	Y	Y	Y	Y	Y	Y	Y	Y	N	Y	High
Prem V, Sahoo RC, Adhikari P. (2013) [49]	Y	Y	Y	U	Y	Y	Y	Y	Y	Y	Y	High
Grams ST, Ono LM, Noronha MA, Schivinski CI, Paulin E. (2012) [50]	Y	Y	Y	Y	Y	Y	Y	Y	N	Y	Y	High
Thomas MJ, Simpson J, Riley R, Grant E. (2010) [51]	Y	Y	Y	Y	Y	Y	U	Y	N	Y	Y	Moderate
Lewis LK, Williams MT, Olds T. (2007) [52]	Y	Y	Y	Y	Y	Y	Y	Y	Y	Y	Y	High
Crowe J, Reid WD, Geddes EL, O’Brien K, Brooks D. (2005) [53]	Y	Y	Y	U	N	Y	Y	Y	N	Y	Y	Moderate
%	84	100	93	93	93	98	77	72	51	91	100	
Y. Yes, N. No, U. Unclear.												

JBI Critical Appraisal Checklist for Systematic Reviews and Research Syntheses: Q1. Was the review question clearly and explicitly stated? Q2. Were the inclusion criteria appropriate for the review question? Q3. Was the search strategy appropriate? Q4. Were the sources and resources used to search for studies adequate? Q5. Were the criteria for appraising studies appropriate? Q6. Was critical appraisal conducted by two or more reviewers independently? Q7. Were there methods to minimize errors in data extraction? Q8. Were the methods used to combine studies appropriate? Q9. Was the likelihood of publication bias assessed? Q10. Were recommendations for policy and/or practice supported by the reported data? Q11. Were the specific directives for new research appropriate? A total of 0–3 represents very low-quality score, 4–6 represents low-quality score, 7–9 represents moderate-quality score, and 10–11 represents high-quality score.

**Table 2:** Respiratory muscle training program.

ID	Intervention	Duration	Length of Session, Frequency	Intensity
Ana Machado, et al. (2020) [21]	Inspiratory muscle training	12 weeks	Two sessions per week to five sessions per day. Duration of sessions varied from < 15 min to 2 h.	
Desjardins, M., & Bonilha, H. S. (2020) [22]	Respiratory muscle training/IMT/EMT/ Incentive spirometry	3 and 7 times a week	Five sets of five breaths with a pause between each set.	The threshold pressure was based on the participants’ maximum expiratory pressure (MEP) in the case of EMST and maximum inspiratory pressure (MIP) in the case of IMST. (75% MEP and 80% MIP).
Zhang, X.; et al. (2020) [18]	Respiratory muscle training	3 weeks	Three repetitions per week for more than 20 min per day.	
van de Wetering-van Dongen, et al. (2020) [20]	Inspiratory muscle training	4 to 12 weeks	Training frequency of 5 sets and 5 repetitions 6 days a week complemented by postural techniques.	A total of 75% MEP.
Lee, E.N; Kim, M.J. (2018) [28]	Respiratory muscle training		A total of 10 sessions for 90 min twice a week to daily.	
Zahra Sadek, et al. (2018) [23]	Inspiratory muscle training	6 to 12 weeks.	Three, six, or seven times per week for 15 to 30 min.	Intensity ranging from 30% to 60%. We can consider 60% of PImax to be the best intensity to apply.
Nepomuceno Jr BRV, et al. (2016) [59]	Inspiratory muscle training	11 weeks	A total of 1 series of 30 breaths repeated twice daily.	Inspiratory load of approximately 50% of maximal inspiratory pressure (MIP).
Gomes Neto, M. et al. (2016) [33]	Inspiratory muscle training		A total of 3 to 5 series for 20–30 min with 10 repetitions.	A total of 15% to 60% of maximal inspiratory pressure.
Chelsea E. Machpherson, Clare C. Bassile (2016) [34]	Inspiratory muscle training		A total of 10 min per day 2 to 3 times a day, which could be progressively increased.	
Klefbeck and Hamrah Nedjad, 2003 in Campbell E.; et al (2015) [55]	Inspiratory muscle training	10 weeks	A total of 3 sets of 10 repetitions twice every second day (n = 7).	
Martín-Valero, R., et al. (2015). [37]	Respiratory muscle training and inspiratory muscle protocol	8-week training	A total of 5 d/week for 30 min/d.	Started with an intensity of 30% of PImax and increased 2 cm H2O.
Martín-Valero, R., et al. (2015). [37]	Program of conventional neurological rehabilitation that was supplemented with an inspiratory muscle training protocol with a threshold value	6 weeks	A total of 2 sessions/d 6 times/week with each session lasting 15 min.	Started with an initial load of 40% until they reached 60% of the Pimax.
Martín-Valero, R. et al. (2014) [44]	Respiratory muscle training	10 weeks to 3 months	Frequency of 7 d/week with 1 or 2 daily sessions consisting of 3 sets with 10 or 15 repetitions per set.	A total of 0–60% of the subject’s maximum expiratory pressure.

**Table 3:** Impact of interventions included in systematic reviews on compromised ventilation.

Citation	Oxygen Saturation	RR/HR/ I:E Ratio	VM /VC	Dyspnea	Spirometry/Pulmonary Function	Peak Flow Rate	Pimax/Pemax	Diffusion	Respiratory Muscle Strength	Walking Test	Quality of Life	Duration of Ventilation/Duration of Weaning	Chest Expansion (Manual Physical Examination)	Decreased Respiratory Comp.
McMahon, L., et al. (2021) [11]		X	X	X	X	X			X					
Wu F, Liu Y, et al. (2020) [12]			X		X	X	X			X				x
Ying Yang, et al. (2020) [13]					X					X				
Chorattas A; et al. (2020)” [14]	X	x		X	X		X			X	X			
GM Monsur Habib; et al. (2020) [15]				X						X	X			
Michael Hindelang, et al. (2020) [16]				X							X			
Dunja Kokotovic, et al. (2020) [17]														x
Xintong Zhang, et al. (2020) [18]	x	x			X		X		X	X	X		X	
Rosa Silva JP, et al. (2020) [19]	X				X		X		X		X			
van de Wetering-van Dongen, et al. (2020) [20]	X	X	X	X	X	X	X		X					
Machado A, et al. (2020) [21]	X	X		X	X					X				
Desjardins, et al. (2020) [22]			X		X	X	X		X					
Azambuja, et al. (2020) [23]				X	X	X	X	X	X	X	X			
Bolger JC, et al. (2019) [24]	X				X		X	X		X	X			
Yu X, Li X, et al. (2019) [25]					X	X				X				
Wang YQ, et al. (2019) [26]	X			X	X	X	X		X	X				
Katz, S., et al. (2018) [27]				X	X	X		X						
Sadek Z, et al. (2018) [23]				X			X	X		X	X			
Eun Nam Lee, Moon Ja Kim (2018) [59]				X	X		X			X				
Vorona S, et al. (2018) [29]			X				X		x			X		
Torres-Sánchez I, et al.(2017) [30]				X	X						X			
Narayanan AL, et al. (2016) [31]														
Volpe MS, et al. (2016) [32]					X		X					X		
Gomes Neto M, et al. (2016) [33]	X	X	X	X	X	X	X	X						
Macpherson CE, Bassile CC (2016) [34]	X	X	X	X	X	X	X				X			
Mohammed J, et al. (2016) [35]	X	X												
Elkins M, Dentice R. (2015) [36]		X							X			X		
Martín-Valero et al. (2015) [37]				X	X	X	X		X	X				
Mans, C. M., Reeve, J. C., & Elkins, M. R. (2015) [38]	X				X	X	X		X	X	X	X		
Montemezzo D, et al. (2014) [39]	X	X	X				X			X		X		
Neves LF, Reis MH, et al. (2014) [40]				X	X		X	X		X				
Snowdon D, Haines TP, Skinner EH. (2014) [41]												X		X
Osterling K, et al. (2014) [42]		X	X	X		X	X					X		
Jácome, C; Marques, A. (2014) [43]					X					X	X			
Martín-Valero, R., et al. (2014) [44]				X	X		X		X	X	X			
Singh F, Newton RU, et al. (2013) [45]					X			X		X	X			
O’Doherty AF, et al. (2013) [46]	X							X		X				
Silva IS, Fregonezi GA, et al.(2013) [47]				X	X	X	X							
Smart NA, Giallauria F, Dieberg G. (2013) [48]							X	X		X	X			
Prem V, Sahoo RC, Adhikari P. (2013) [49]		X			X			X			X			
Grams ST, et al. (2012) [50]					X		X						X	
Thomas MJ, et al. (2010) [51]				X					X					
Lewis LK, Williams MT, Olds T. (2007) [52]	X	X	X	X	X			X	X	X		X	X	
Crowe J, et al. (2005) [53]				X	X				X	X	X			

**Table 4:** Summary of evidence.

Interventions	Included Systematic Reviews	Outcomes								
		Oxygen Saturation	Dyspnea	I:E Ratio and FR	Spirometry	Peak Flow	Pimax and Pemax	Diffusion	Respiratory Muscle Strength	Walking Test
Inspiratory muscle training	McMahon, L. et al. (2021) [11] Wu, F. et al. (2020) [12] Van de Wetering-van Dongen et al. (2020) [20] Azambuja, A. et al. (2020). [23] Xintong Z et al. (2020) [18] Bolger, J.C. et al. (2019) [24] Sadek, Z. et al. (2018) [23] Eun Nam Lee, et al. (2018) [59] Gomes Neto M, et al. (2016). [33] Mans, C. M., et al. (2014) [38] Neves, L.F. et al. (2014) [40] Martín-Valero, R. et al. (2014) [44] Silva IS, et al. (2013) [47] Montemezzo D, et al. (2014) [39] Smart N.A. et al. (2013) [48] Crowe J, et al. (2005) [53]									
Breathing control (relaxed breathing, pursed-lip breathing, and diaphragmatic breathing exercises)	Lewis LK. et al. (2007) [52] Grams ST, et al. (2012) [50] Prem V. et al. (2013) [49] Wang YQ. et al. (2019) [26] Ying Yang, et al. (2020) [13] Chorattas A. et al. (2020) [14]									
Respiratory expansion exercise (deep breathing exercise)	Grams ST, et al. (2012) [50] Wang YQ. et al. (2019) [26] Chorattas A. et al. (2020) [14]									
Thoracic expansion exercises	Grams ST, et al. (2012) [50] Wang YQ. et al. (2019) [26] Chorattas A. et al. (2020) [14]									
Positioning to optimize ventilation	Katz, S., et al. (2018) [27]									

Green area represents overall effective; Yellow area represents no overall effect or difference compared to a control treatment; White area represents no data reported. All significant effects identified in systematic reviews had a small magnitude. The variability among the included studies precludes a detailed evaluation of effect sizes as well as separate comparisons against the individual interventions. The transposition of this evidence into practice should be based on the assumption that each of these interventions was effective against placebo or some other form of acceptable clinical intervention.

**Table 5:** Summary of findings.

Outcomes	Impact	Nº of Participants (Studies)	Certainty of the Evidence (GRADE)
Inspiratory Muscle Training			
Oxygen saturation	Effective improvement with small positive effects *	2740 (10 reviews)	⨁⨁⨁◯ MODERATE due to inconsistency (due to significant heterogeneity)
Dyspnea (several instruments were used to measure this outcome)	Effective improvement with moderate positive effects **	2740 (10 reviews)	⨁⨁⨁⨁ HIGH
Respiratory rate	Effective improvement with small positive effects *	371 (1 review)	⨁⨁⨁⨁ HIGH
Inspiration:expiration ratio (I:E)	Effective improvement with small positive effects *	371(1 review)	⨁⨁⨁⨁ HIGH
Respiratory muscle strength	Effective improvement with moderate positive effects **	2541 (6 reviews)	⨁⨁⨁⨁ HIGH
Maximum expiratory pressure (MEP)/ maximum inspiratory pressure (MIP)	Effective improvement with moderate positive effects **	2656 (8 reviews)	⨁⨁⨁⨁ HIGH
Pulmonary function testing (several instruments were used to measure this outcome)	Effective improvement with moderate positive effects **	4414 (10 reviews)	⨁⨁⨁⨁ HIGH
Peak expiratory flow	Effective improvement with moderate positive effects **	1619 (5 reviews)	⨁⨁⨁⨁ HIGH
6 min walking test (6 MWT)	Effective improvement with small positive effects *	2709 (9 reviews)	⨁⨁⨁◯ MODERATE due to inconsistency (due to significant heterogeneity)
Diffusion	Effective improvement with moderate positive effects **	1369 (3 reviews)	⨁⨁⨁◯ MODERATE due to inconsistency (due to significant heterogeneity)
Quality of life	Effective improvement with small positive effects *	958 (2 reviews)	⨁⨁⨁⨁ HIGH
Postoperative outcomes (respiratory as primary endpoint)	Effective improvement with moderate positive effects **	708 (1 review)	⨁⨁◯◯ LOW
Adverse events	Effective improvement with small positive effects *	330 (1 review)	⨁⨁⨁◯ MODERATE due to inconsistency (due to significant heterogeneity)
Inspiratory muscle training in critical care			
Duration of postoperative mechanical ventilation	Effective improvement with small positive effects *	330 (1 review)	⨁⨁⨁◯ MODERATE due to inconsistency (due to significant heterogeneity)
Rapid shallow breathing index	Effective improvement with moderate positive effects **	105 (1 review)	⨁⨁⨁⨁ HIGH
Maximum expiratory pressure (MEP)/ maximum inspiratory pressure (MIP)	Effective improvement with moderate positive effects **	1846 (3 reviews)	⨁⨁⨁⨁ HIGH
Length of stay	Effective improvement with small positive effects*	291 (1 review)	⨁⨁⨁⨁ HIGH
Postoperative pulmonary complications	Effective improvement with moderate positive effects **	291 (1 review)	⨁⨁⨁⨁ HIGH
Time to extubation	Effective improvement with moderate positive effects **	291 (1 review)	⨁⨁⨁⨁ HIGH
Reduced duration of weaning from mechanical ventilation	Effective improvement with moderate positive effects **	1185 (1 review	⨁⨁⨁⨁ HIGH
Ventilator weaning duration	Effective improvement with small positive effects *	479 (2 reviews)	⨁⨁⨁⨁ HIGH
Success rate in weaning IMV	Effective improvement with small positive effects *	523 (2 reviews)	⨁⨁⨁⨁ HIGH
Duration of mechanical ventilation	Effective improvement with small positive effects *	305 (1 review)	⨁⨁⨁⨁ HIGH
Reintubation	Effective improvement with small positive effects *	117 (1 review)	⨁⨁⨁⨁ HIGH
Breathing Control (relaxed breathing, pursed-lip breathing, and diaphragmatic breathing exercises)			
Pulmonary function testing (several instruments were used to measure this outcome)	Effective improvement with moderate positive effects **	963 (6 reviews)	⨁⨁⨁⨁ HIGH
Expiratory flow rate	Effective improvement with small positive effects *	285 (6 reviews)	⨁⨁⨁◯ MODERATE due to inconsistency (due to significant heterogeneity)
Respiratory muscle strength	Effective improvement with moderate positive effects **	132 (1 review)	⨁⨁⨁⨁ HIGH
Dyspnea (several instruments were used to measure this outcome)	Effective improvement with moderate positive effects **	803 (2 reviews)	⨁⨁⨁◯ MODERATE due to inconsistency (due to significant heterogeneity)
Respiratory rate	Effective improvement with moderate positive effects **	539 (2 reviews)	⨁⨁⨁◯ MODERATE due to inconsistency (due to significant heterogeneity)
Abdominal excursion/diaphragm excursion	Effective improvement with moderate positive effects **	285 (1 review)	⨁⨁⨁◯ MODERATE due to inconsistency (due to significant heterogeneity)
Oxygen saturation (Sat O2) (several instruments were used to measure this outcome)	Effective improvement with moderate positive effects **	285 (1 review)	⨁⨁⨁◯ MODERATE due to inconsistency (due to significant heterogeneity)
Diffusion	Effective improvement with moderate positive effects **	254 (1 review)	⨁⨁⨁◯ MODERATE due to inconsistency (due to significant heterogeneity)
Walk test (MWT) (several instruments were used to measure this outcome)	Effective improvement with moderate positive effects **	1961 (3 reviews)	⨁⨁⨁◯ MODERATE due to inconsistency (due to significant heterogeneity)
Postoperative pulmonary complications (PPCs)	Effective improvement with moderate positive effects **	1402 (2 reviews)	⨁⨁⨁⨁ HIGH
Respiratory expansion exercise (deep breathing exercise)			
Pulmonary function testing (several instruments were used to measure this outcome)	Effective improvement with moderate positive effects **	1270 (2 reviews)	⨁⨁⨁⨁ HIGH
Dyspnea	Effective improvement with moderate positive effects **	518 (1 review)	⨁⨁⨁◯ MODERATE due to inconsistency (due to significant heterogeneity)
Respiratory muscle strength	Effective improvement with moderate positive effects **	132 (1 review)	⨁⨁⨁⨁ HIGH
6 min walk test (6 MWT)	Effective improvement with moderate positive effects **	1270 (1 review)	⨁⨁⨁⨁ HIGH
Postoperative pulmonary complications (PPCs)	Effective improvement with moderate positive effects **	1402 (2 reviews)	⨁⨁⨁⨁ HIGH
Thoracic expansion exercises			
Pulmonary function testing (several instruments were used to measure this outcome)	Effective improvement with moderate positive effects **	1402 (2 reviews)	⨁⨁⨁⨁ HIGH
Respiratory muscle strength	Effective improvement with moderate positive effects **	132 (1 review)	⨁⨁⨁⨁ HIGH
6 min walk test (6 MWT)	Effective improvement with moderate positive effects **	1270 (1 review)	⨁⨁⨁⨁ HIGH
Postoperative pulmonary complications (PPCs)	Effective improvement with moderate positive effects **	1402 (2 reviews)	⨁⨁⨁⨁ HIGH
Positioning to optimize ventilation			
Pulmonary function testing (several instruments were used to measure this outcome)	Effective improvement with small positive effects *	1 review (38 studies)	⨁⨁◯◯ LOW
PEF	Effective improvement with small positive effects *	1 review (13 studies)	⨁⨁◯◯ LOW
FRC	Effective improvement with small positive effects *	1 review (5 studies)	⨁⨁◯◯ LOW
Total Lung Capacity	no evidence	1 review (2 studies)	⨁⨁◯◯ LOW
Residual Volume	no evidence	1 review (2 studies)	⨁⨁◯◯ LOW
Pimax/PE max	Effective improvement with small positive effects *	1 review (6 studies)	⨁⨁◯◯ LOW
DLCO (Diffusion capacity)	Effective improvement with small positive effects *	1 review (7 studies)	⨁⨁◯◯ LOW

* The effect was interpreted as small positive if it was less than 0.40. ** The effect was interpreted as moderate positive if it was greater than 0.40 and less than 0.80. GRADE Working Group grades of evidence: High certainty (⨁⨁⨁⨁) represents that we were very confident that the true effect lies close to that of the estimate of the effect. Moderate certainty (⨁⨁⨁◯) represents that we were moderately confident in the effect estimate. The true effect is likely to be close to the estimate of the effect, but there is a possibility that it is substantially different. Low certainty (⨁⨁◯◯) represents that our confidence in the effect estimate was limited. The true effect may be substantially different from the estimate of the effect. Very low certainty (⨁◯◯◯) represents that we had very little confidence in the effect estimate. The true effect is likely to be substantially different from the estimate of effect.

## Data Availability

The present study was carried out according to the guidelines of the Declaration of Helsinki and was approved by an ethics committee.

## Supplementary Materials

The following supporting information can be downloaded at: https://www.mdpi.com/article/10.3390/ijerph20075239/s1, Supplementary S1: Search Stategy; Supplementary S2: Data extraction.
