# Supplementary material for: Effectiveness of Nonpharmacological Interventions in the Field of Ventilation: An Umbrella Review
Source: Int J Environ Res Public Health. 2023 Mar 23;20(7):5239. doi: 10.3390/ijerph20075239 (PMC10093871; doi:10.3390/ijerph20075239)
Supplement: Supplementary file 1 [file ijerph-20-05239-s001.zip › ijerph-2199807-supplementary.pdf]

Supplementary S1: Search Stategy

SCOPUS - 481 results

Web of Science – 406 results JBI – 0 results

Ebsco - CINAHL Complete, CINAHL Plus with Full Text, MedicLatina, MEDLINE with Full Text, MEDLINE, ERIC – 202 results

Cochrane Reviews – 9 results (Includes EMBASE) & PubMed

Search	Query	Records retrieved
#1	TI ("Thorax expansion" or "Continuous positive airway pressure titration" or "Eucapnic voluntary hyperventilation challenge" or "Physiotherapy of chest" or "Active cycle of breathing technique") AND TI (review or meta-analysis or metareview or meta-review or meta review or literature review) NOT TI "airway clearance"	
#2	TI (“Pulmonary rehabilitation” or “Oscillating positive expiratory pressure physiotherapy” or “Respiratory therapy” or “Physiotherapeutic breathing exercise” or “Diaphragmatic breathing exercises” or “Relaxed breathing” or “Breathing exercise, blow bottle” or “Respiratory expansion exercises” or “Apical expansion exercises” or “Basal expansion exercises” or “Lower lateral costal expansion exercises” or “Thoracic expansion exercises” or “Lower thoracic expansion exercises” or “Upper thoracic expansion exercises” or “Breathing control” or “Inspiratory muscle training” or “Incentive spirometry”) AND TI (review or meta-analysis or metareview or meta-review or meta review or literature review) NOT TI "airway clearance"	
#3	#1 AND #2	1089
No Limited to #date, language limits: etc.#		823

Supplementary S2: Data extraction

Quality of the evidence (JBI Effectiveness)	Studies	Author /year	Country	Study design	Objectives	Study Population	Outcome measures	Types of interventions	Results
1.b	Nonpharmacological interventions for respiratory health in Parkinson's disease: A systematic review and meta-analysis.	McMahon, L., Blake, C., & Lennon, O. (2021)	Ireland	Systematic review and meta-analysis	The purpose of this review is to systematically identify randomized and non-randomised controlled trials that report non-pharmacological interventions used to improve respiratory metrics for people with PD. This will allow strategies currently employed to be identified and their effectiveness in improving respiratory	included 371 participants with PD, 210 were male and 161 were female	Inspiratory Muscle Strength, Expiratory Muscle Strength, Sniff Nasal Inspiratory Pressure, FVC, FEV1, FEV1/FVC, Peak Expiratory Flow, Lung volume measurements, Peak Cough Flow, and the Modified Borg Scale for dyspnoea.	Respiratory muscle strength training (inspiratory and/or expiratory); incentive spirometry and singing	Best-evidence synthesis identified level 1 evidence supporting nonpharmacological interventions for improving peak cough flow and perceived dyspnoea. No studies were identified reporting outcomes of respiratory rate, inspiration:expiration ratio or respiratory morbidity or mortality in PD. Nonpharmacological interventions improved respiratory muscle strength and peak expiratory flow in PD. Meta-analyses conducted demonstrate efficacy for non-pharmacological

					function to be better elucidated.				interventions in improving respiratory muscle strength (both inspiratory and expiratory) and peak expiratory flow rates.
**1.a**	Respiratory muscle training improves strengthand decreases the risk of respiratory complications in stroke survivors: a systematic review and meta-analysis	Wu F, Liu Y, Ye G, Zhang Y (2020)	China	Systematic Review of Randomized Controlled Trials	To evaluate the effects of respiratory muscle training in a population of stroke patients	Patients were included regardless of gender, age or the time period since the 62 occurrence of the stroke, namely the acute/sub-acute or chronic phases. A total of 308 patients from 9 RCT studies	maximal inspiratory pressure, maximal expiratory pressure , forced expiratory volume in 1 s,forced vital capacity, peak expiratory flow, 6-minute walk test and decreased respiratory complications	Respiratory muscle training, Frequency and duration : "Patients underwent training for 20-40 min or for between 50 and 100 repetitions, 3 to 14 times a week for 3 to 8 weeks."	Respiratory muscle training improved post-stroke muscle strength and the benefits were carried over for up to 12-weeks, including improved lung function, walking capacity and a reduced risk of respiratory impediments.

**1.a**	The effects of pursed lip breathing combined with diaphragmatic breathing on pulmonary function and exercise capacity in patients with COPD: a systematic review and meta analysis	Ying Yang , Liuyi Wei , Shizhen Wang , Li Ke , Huimin Zhao , Jing Mao , Jie Li & Zongfu Mao (2020)	China	a systematic review and meta-analysis, Randomized controlled trials (RCTs)	This systematic review aimed to investigate the effects of PLB combined with DB on pulmonary function and exercise capacity in patients with COPD.	participants were diagnosed as COPD; (2) randomized controlled trial (RCT) study design;	pulmonary function: the forced vital capacity, FVC, FEV1), and the ratio of these two measurements (FEV1/FVC) A 6-min walk test (6MWT) was adopted for measuring exercise capacity according	the intervention was pursed lip breathing (PLB) and combined with DB diaphragmatic breathing	The results of pooled analysis indicated that PLB combined with DB provided higher gains in FEV 1, FVC, FEV1 /FVC, and 6MWT. This study suggested that PLB combined with DB was favorable for improving pulmonary function of patients with COPD.

**1 b.**	Home-Based Educational Programs for Management of Dyspnea - A Systematic Literature Review	Aristides Chorattas; Evridiki Papastavrou; Andreas Charalambous; Christiana Kouta (2020)	Cyprus	Systematic review	Identify whether there are any home-based educational programs for the support of patients with dyspnea due to lung cancer run by nurses, as well as their effectiveness in managing patients' dyspnea outside the hospital environment	patients with dyspnea (7 research studies): 1- 71 lung cancer or malignant pleural mesothelioma patients receiving chemo/radiotherapy; 2- 106 COPD patients from a certain geographical region; 3 and 4- 34 and 177 COPD patients; 5- restrictive lung disease and COPD patients; 6 and 7 - 32 and 32 COPD patients	Exercise capacity, mMRC scale, QoL and phychological state; MRC dyspnea scale, lung function with spirometry, dyspnea on exertion with mBorg, HRQOL with CRDQ; SGRQ, knowledge of illness, FVC/FEV, Pimax/Pemax, FEV and FVC. dyspnea level with Baseline Dyspnea Index (BDI), functional capacity measured with the 6MWT; Vital signs seen in weeks 1-3-6-9-12. CRDQ and self-efficacy on weeks 1-6-12	pulmonary rehabilitation (PR), which included either breathing retraining (diaphragmatic breathing, inspiratory and/or expiratory muscle training, pursed-lip breathing, respiratory muscle stretching calisthenics) or breathing exercises or exercise training (stretching, walking, stairs climbing, upper and lower aerobic)	The results of the studies showed significant benefits for the intervention group in improving dyspnea not only in relation to the initial assessment but also compared to the control group.<br>In the study by Olivier et al, improvement of six-minute walk test (6MWT) and a test of 10 chair stands (10CS) was shown without decreasing dyspnea significantly.<br>In another two studies, there were no changes in the Pulmonary Function Test even though dyspnea improved and in one there was increased physical exertion in the intervention group.<br>In the study by Akinci and Olgun, arterial blood gases improved in both groups but with statistical importance only in

									the intervention group. In the study conducted by Kagaya et al where both groups received the intervention, it showed that there were improvements either in the same parameters (PImax, PEmax, 6MWD) or in different ones.
**1.b**	Systematic review of clinical effectiveness, components and delivery of pulmonary rehabilitation in low-resourse settings	GM Monsur Habib; Roberto Rabinovich ; Kalyani Divgi; Salahuddin Ahmed; Samir Kumar Saha; Sally Singh; Aftab Uddin; Md.	UK	Systematic review, RCT and clinical controlled trials	Review the effectiveness , components and mode of delivery of PR in low-resource settings.	Study participants were COPD patients of varying degree of severity in all the trials except one which recruited people with pulmonary impairment after TB (PIAT). Total number of enrolled participants was 661 of which	Our primary outcomes were between-group difference in functional exercise capacity (e.g. 6-MWT80–82) and HRQoL (e.g. SGRQ83,84). We also included breathlessness (e.g. mMRC Dyspnoea score)	All interventions included exercise programme, e upper limb exercise, breathing exercices (including IMT), pursed-lip breathing, diaphragmatic breathing, knowledge, psychological interventions	Breathlessness was measured in 11 studies of which 9 studies showed significant positive changes and 2 studies (1 at moderate RoB) showed no changes after intervention. None of the studies reported negative effects after the intervention. Although not

		Nazim Uzzaman; Hilary Pinnock (2020)				COPD and PIAT were 83% and 17%, respectively.		(CBT, relaxation), coping strategies, nutrition, self-management, social support, pharmacological optimization	described in detail, the other common component was breathing exercises included in eight studies. Along with the exercise, patient education was provided in ten studies and skills (such as inhaler technique and airway clearance) were included in seven studies.
**1.a**	Effectiveness of non-pharmacological COPD management on health-related quality of life - a systematic review	Michael Hindelang, Florian Kirsch & Reiner Leidl (2020)	Germany	Systematic review	Compile the current evidence on the HRQoL effects of non-farmacological interventions consisting of pulmonary rehabilitation, physical activity, and training compared with usual care or no intervention in COPD according to the main	Patients whose main disease was COPD (sample sizes ranged from 30 to 155, total of 1059 patients)	Quality of life, EQ-5D, SF-36, SGRQ, George Respiratory questionnaire, mMrc, COPD Assessment test, CAT, Quality adjusted life year, CCQ, COPD anxiety questionnaire, SGRQ-C, HRQOL, HRQL,	Non-medicinal, non-pharmacological, pneumologic rehabilitation, training, physical activity, breath therapy, relaxation, non-invasive ventilation, ambulatory oxygen therapy, short-burst oxygen therapy, long term oxygen therapy, extracorporeal membrane oxygenation, lung transplant, smoking cessation, non-	he studies strongly support the positive effects on HRQoL of non-pharmacological programs in COPD, which mainly include pulmonary rehabilitation with components of physical activity, education or counselling-based training programs, or breathing exercises. To improve COPD care, further measures are needed to increase the accessibility and applicability of non-

					components of the intervention and their intensity			invasive positive pressure ventilation, telemedicine, education, pulmonary rehabilitation, bullectomy, invasive ventilation, immunization, lung volume reduction	pharmacological treatment programs. In addition, non-pharmacological treatments should be tailored to the needs of the patient to best improve their HRQoL All three studies with breathing exercises as the intervention showed significant and clinically relevant improvements in HRQoL This type of intervention is easier to carry out in patients' daily lives than training programs that require bicycles or treadmills.

**1.b**	The effect of a postoperative respiratory and mobilization interventions on postoperative complications following abdominal surgery: a systematic review and meta-analysis	Dunja Kokotovic, Adam Berkfors, Ismail Gögenur, Sarah Ekeloef, Jakob Burchart (2020)	Denmark	Systematic review and meta-analysis	The purpose of this systematic review and meta-analyses was to investigate whether postoperative respiratory interventions and mobilization interventions compared with usual care can prevent postoperative complications following abdominal surgery.	The participants (P) of interest were patients (≥18 years of age) undergoing intraabdominal gastrointestinal surgery. Patients with preexisting pulmonary/respiratory conditions were also included.	Primary outcome measures: postoperative complications including all pulmonary complications (pneumonia, atelectasis, pleural efusion, bronchitis), surgical complications (reoperations, wound infections, reoperations etc.), and medical complications (urinary tract infections, cardiovascular complications, sepsis, etc.). Secondary outcome measures: length of hospital stay, mortality, and possible side efects to physiotherapeutic interventions.	The interventions included respiratory interventions with and without adjacent breathing devices such as CPAP (Continuous positive airway pressure), EPAP (Expiratory positive airway pressure), BiPAP (Bilevel positive airway pressure), NIV (Non-invasive ventilation), IPPB (intermittent positive pressure breathing), IPAP (inspiratory positive airway pressure), spirometry, and PEP (positive expiratory pressure), muscle training, or structured breathing exercises.	Pulmonary complications were addressed in 25 studies containing 2068 patients. Twenty-three studies were included in the meta-analyses. Patients predominantly underwent open elective upper abdominal surgery. Postoperative respiratory interventions consisted of expiratory resistance modalities (CPAP, EPAP, BiPAP, NIV), assisted inspiratory fow modalities (IPPB, IPAP), patient-operated ventilation modalities (spirometry, PEP), and structured breathing exercises. Meta-analyses found that ventilation with high expiratory resistance (CPAP, EPAP, BiPAP, NIV) reduced the risk of

									pulmonary complications with OR 0.42 (95% CI 0.18–0.97, p=0.04, I^2= 0%) compared with usual care, however, the trial sequential analysis revealed that the required information size was not met. Neither postoperative assisted inspiratory fow therapy, patient-operated ventilation modalities, nor breathing exercises reduced the risk of pulmonary complications.

**1.a**	Can inspiratory muscle training benefit patients after stroke? A systematic review and meta-analysis of randomized controlled trials	Xintong Zhang, Yu Zheng, Yini Dang, Lu Wang, Yihui Cheng, Xiu Zhang, Mao Mao, Xiao Lu (2020)	China	Systematic review and Meta-analysis	investigate the effects of inspiratory muscle training in post-stroke patients and to explore the effective training protocol.	Thirteen enrolled trials randomized a total number of 373 patients either into the experimental group (n = 189) or the control group (n = 184)	Forced vital capacity, forced expired volume in 1 second, 6-minute walk test, maximum inspiratory pressure, inspiratory muscle endurance, pulmonary infection incidence	Inspiratory muscle training, assisted exercises, or abdominal strengthening and breathing exercises	Meta-analysis conducted in 8 out of 13 trials revealed evidence for beneficial effects of inspiratory muscle training on forced vital capacity (MD: 0.47, 95% CI: 0.28–0.66), forced expired volume in 1 second (MD: 0.26, 95% CI: 0.18–0.35), 6-minute walk test (MD: 52.61, 95% CI: 25.22–80.01), maximum inspiratory pressure (MD: 18.18, 95% CI: 5.58–30.78), inspiratory muscle endurance (MD: 19.99, 95% CI: 13.58–26.40), and pulmonary infection incidence (RR: 0.11, 95% CI: 0.03–0.40). The effective inspiratory muscle training protocol was suggested by subgroup analysis with three repetitions per week and more than 20minutes per day for three weeks. Inspiratory muscle training can be

									considered as an effective intervention for improving pulmonary function and cardiopulmonary endurance, and reducing pulmonary infection incidence in patients after stroke."
**1.b**	Quality of life and functional independence in amyotrophic lateral sclerosis: A systematic review.	Rosa Silva JP, Santiago Júnior JB, Dos Santos EL, de Carvalho FO, de França Costa IMP, Mendonça DMF (2020)	Brazil	Systematic review	analyze treatment protocols and their outcomes from clinical trials with focus on ALS rehabilitatio n that evaluated the effects on quality of life and functional independenc e from their intervention process.	The population should be composed of ALS patients, with no phenotype, age or gender restriction. Eleven studies	Quality of life was evaluated. respiratory function, vital capacity improvement, nocturnal oxygen saturation	Bipap support, Non-invasive nigh-time ventilation, Active inspiratory exercise program	All studies proposed interventions aimed at minimizing the altera tions resulting from the progression of the disease in the communica tion, psychological, and respiratory aspects, being the latter the most explored in most articles. Strategies such as Inspiratory Muscular Training (IMT) associated with the use of Non-Invasive Mechanical

									Ventilation (NIMV), and intermittent positive two-way air pressure (Bipap) were used to demonstrate benefits to patients.
**1.a**	The Effects of Respiratory Training in Parkinson's Disease: A Systematic Review.	van de Wetering-van Dongen, V. A., Kalf, J. G., van der Wees, P. J., Bloem, B. R., & Nijkrake, M. J. (2020)	Netherlands	Systematic review	The purpose of this systematic review is to review the efficacy of different respiratory training interventions in PD.	persons with PD in Hoehn & Yahr stage I to III.	incentive spirometry, respiratory function tests , FVC, FEV1, MVV, MIP and MEP	inspiratory muscle strength training (IMST), expiratory muscle strength training (EMST), air stack ing, breath-stacking, incentive spirometry and postural training on respiratory muscle strength, swallowing safety,	Respiratory training shows positive effects and should be considered when people with PD experience respiratory dysfunction. The overall conclusion of this systematic review is that all respiratory training interventions show positive effects in people with PD, underlining that respiratory training should be considered as a pos sible treatment option for people with PD.

**1.a**	Design of pulmonary rehabilitation programmes during acute exacerbations of COPD: a systematic review and network meta-analysis.	Machado A, Matos Silva P, Afreixo V, Caneiras C, Burtin C, Marques A.(2020)	Portugal	systematic review and network meta-analysis.	This systematic review aimed to systematise the different designs used to deliver pulmonary rehabilitation during acute exacerbations of COPD (AECOPD) and explore which ones are the most effective.	total of 42 studies were included in this review and 25 were used for meta-analysis. In total, 3569 patients with a mean age of 69.1 years	respiratory function tests , FVC, FEV1, SO2, 6MWT, Dyspnoea symptoms: modified Borg scale, Length of hospitalisation: number of days	Education and psychosocial support was the most used component, followed by breathing techniques(i.e. breathing control and/or airway clearance techniques), exercise training as part of the intervention. Other additional components used were e positive expiratory pressure therapy performed with noninvasive ventilation or devices (n=3) , home diaries, respiratory muscle training	Pulmonary rehabilitation is a safe intervention during AECOPD. Exercise, breathing techniques, and education and psychosocial support seem to be the core components for implementing pulmonary rehabilitation during AECOPD. Overall pooled results showed that combining exercise with breathing techniques is the most effective intervention to improve exercise capacity during AECOPD and the only design that results in improvements above the minimal clinically important difference of 30 m established for the 6MWT
**1.b**	The Impact of Respiratory Exercises on Voice Outcomes: A Systematic	Desjardins, M., & Bonilha, H. S. (2020)	South Carolina	Systematic Review	The goal of the present review is to determine the state of the evidence	vocally healthy participants; these were professional classical sing ers	Spirometry measures . Maximum respiratory pressure measures MIP	expiratory muscle strength training; inspiratory muscle strength training;	EMST, IMST, incentive spirometry, abdominal directives, and stimulation training

	Review of the Literature				regarding the effectiveness of respiratory intervention s to improve respiratory and voice outcomes	and classical singing students.	and MEP indirectly measure respiratory muscle strength by using a hand-held pressure meter or a manometer	incentive spirometry; isocapnic hyperpnea; “easy breathing”; with abdominal breath support.	focusing on abdominal support all had a positive effect on at least one respiratory outcome. EMST improves MEP EMST interventions improved MEP in all seven studies in which it was assessed
**1.a**	Inspiratory Muschle Training in Patients with Heart Failure: What is new? Systematic review and meta-analysis	Azambuja, A., de Oliveira, L. Z., & Sbruzzi, G. (2020)	Brazil	Systematic review and meta-analysis	The purpose of this study was to review the effects of IMT on respiratory muscle strength, functional capacity, pulmonary function, quality of life, and dyspnea in patients with HF; IMT isolated or combined with another intervention (combined IMT), the presence of	Patients with heart failure (n=374 - systematic review, n=342 - meta analysis)	respiratory muscle strength (through MIP and maximal expiratory pressure. Secondary outcomes: pulmonary function (through forced vital capacity - FVC, and forced expiratory volume in the first second - FEV1), functional capacity (assessed with 6MWT and VO2 peak), quality of life (Minnesota Living with Heart Failure	10 studies - isolated IMT, 7 with individuals with inspiratory muscle weakness (6 studies with loads of 30% MIP, 1 with 40%, 2 with 60-90%, 1 with 100% for 10 maximal repetitions. Intervention time - 3 studies - 4-6 weeks, 2 studies - 8 weeks, 5 studies - 12 weeks. Then, 4 studies with IMT combined with another intervention. 1 with patients with inspiratory muscle weakness -- 2 studies	Isolated IMT demonstrared an increase in maximal inspiratort pressure (MIP) (25.12 cm H2O, 95% Cl= 15.29 to 34.95), 6-minute walk test (81.18 m; 95% CI = 9.73 to 152.63), maximum oxygen consumption (12 weeks: 3.75 mL/kg/min; 95% Cl = 2.98 to 4.51), and quality of life (−20.68; 95% CI = −29.03 to −12.32). The presence of inspiratory muscle weakness, higher loads and longer intervention times resulted in greated increases in MIP.

					inspiratory muscle weakness, training load, and intervention time were considered		Questionnaire, where lower scores equal higher quality of life) and dyspnea (Borg scale)	combined with aerobic training, 1 with peripheral resistance training, 1 with neuromuscular electrical stimulation. 3 studies with loads of up 30% of MIP, 1 with 60%. Intervention time: 3 studies - 12 weeks, 1 - 8 weeks	IMT combined with another intervention demonstrated an increase only in MIP. Isolated IMT resulted in an increase in inspiratory muscle strength, functional capacity, and quality of life. IMT combined with another intervention resulted only in a small increase in inspiratory strength. Isolated IMT with higher loads can be considered an adjuvant intervention, especially for those who do not adhere to conventional rehabilitation and who have respiratory muscle weakness.
**1.b**	Perioperative prehabilitation and rehabilitation in esophagogastric malignancies: a systematic review	Bolger JC, Loughney L, Tully R, et al.(2019)	Ireland	Systematic Review	The aim of this study was to perform and present a systematic review of publications that focus on the effect of	708 patients with esophageal malignancies, 118 patients with gastrointestinal malignancies	6MWT, lung function, functional lung capacity, VO2max, QoL, inspiratory muscle function, postoperative outcomes (respiratory as	postoperative exercise and multidisciplinary rehabilitation program; preoperative inspiratory muscle training; pre-operative exercise;	Inspiratory muscle training (IMT) consistently showed improvements in functional status preoperatively, with three studies showing improvements in respiratory

					perioperative rehabilitation on improving outcomes following esophageal or gastric cancer surgery		primary endpoint)	postoperative exercise and multidisciplinary rehabilitation; preoperative education, postoperative exercise, postoperative multidisciplinary rehabilitation; preoperative IMT	complications with IMT. Postoperative rehabilitation was associated with improved clinical outcomes.
**1.a**	Pulmonary Rehabilitation for Exercise Tolerance and Quality of Life in IPF Patients: A Systematic Review and Meta-Analysis.	Yu X, Li X, Wang L, et al.. (2019)	China	systematic review and meta-analysis	Te aim of this study is to evaluate the efcacy and safety of pulmonary rehabilitation (PR) in patients with idiopathic pulmonary fbrosis (IPF)	Seven studies (190 participants) were included	6MWD ,Forced vital capacity (FVC%), lung difusing capacity determined by the single-breath technique (DLCO%)	Education and psychosocial support was the most used component, followed by breathing techniques(i.e. breathing control and/or airway clearance techniques), exercise training as part of the intervention.	Tis study suggests that PR may enhance exercise capacity and improve quality of life in IPF patients. Besides, PR may also delay the decline of lung function of patients with IPF.

**1.a**	Impact of breathing exercises in subjects with lung cancer undergoing surgical resection: a systematic review and meta-analysis	Wang YQ, Liu X, Jia Y, Xie J. (2019)	China	Systematic review and meta-analysis	To assess the effects of breathing exercises on postoperative pulmonary complications (PPCs), pulmonary function, 6-min walk distance (6MWD) and the length of hospital stay (LOS) in lung cancer patients undergoing lung surgery.	Total of 1270 lung cancer patients (in the preoperative intervention groups, the sample size ranged from 17–90 patients, the postoperative sample capacity ranged from 66–116 patients, and the perioperative period sample size ranged from 100–160 individuals. The age of subjects during the course of studies ranged from 35–84 years, and most of them were diagnosed with stages I–III of lung cancer.	Predicted forced expiratory volume in 1 s (predicted FEV1%), predicted forced vital capacity (predicted FVC%), FVC and FEV1/FVC ratio, 6MWD,	breathing exercises, such as pursed-lip breathing, abdominal breathing and thoracic breathing exercises. 8 trials: preoperative intervention, 4 trials: postoperative intervention, 4 trials: perioperative. The duration of breathing exercise interventions ranged from 1 week–6 months, and each exercise had a different intervention frequency. Spirometers and balloons were used for assisting the breathing training.	Breathing exercises decreased PPCs, and in addition, the incidence of pneumonia and atelectasis in the postoperative subgroup was reduced. Predicted forced expiratory volume in 1 s (predicted FEV1%), predicted forced vital capacity (predicted FVC%), FVC and FEV1/FVC ratio had improved after breathing exercises, but the changes in FEV1 were not statistically significant. Furthermore, the LOS was significantly decreased, but no improvements were found in 6MWD. The results showed that breathing exercises could improve lung func tion, decrease the incidence of PPCs and LOS in a sample of lung cancer patients undergoing pulmonary surgery.

									The analysis suggests that breathing exercises should be considered as a respiratory rehabilitation programme for lung cancer patients un dergoing lung surgery in clinical practice.
**1 b.**	The effect of body position on pulmonary function: a systematic review.	Katz, S., Arish, N., Rokach, A., Zaltzman, Y., & Marcus, E. L. (2018)	jerusalem , Israel	Systematic Review	This systematic review investigated the influence of body position on lung function in healthy persons and specific patient groups	A total of 43 studies fully met inclusion criteria and were included in the review. Study population of non-mechanically ventilated subjects. Participants aged ≥18 years. English language. Studies assessing lung function using other criteria and those without statistical comparisons of lung function in	Primary outcome measures were forced expiratory volume in 1 s (FEV1), forced vital capacity (FVC, FEV1/FVC), vital capacity (VC), functional residual capacity (FRC), maximal expiratory pressure (PEmax), maximal inspiratory pressure	Position to optimize ventilatio . Standing, sitting, supine, and right- and left-side lying positions were studied	Body position influences the results of PFTs, but the optimal position and magnitude of the benefit varies between study populations. PFTs are routinely performed in the sitting position. We recommend the supine position should be considered in addition to sitting for PFTs in patients with SCI and neuromuscular disease. When treating patients

						different positions, those enrolling individuals < 18 years or on mechanical ventilation,	(PImax), peak expiratory flow (PEF), total lung capacity (TLC), residual volume (RV), and diffusing capacity of the lungs for carbon monoxide (DLCO).		with heart, lung, SCI, neuromuscular disease, or obesity, one should take into consideration that pulmonary physiology and function are influenced by body position.
**1.a**	Best mode of inspiratory muscle training in heart failure patients: a systematic review and meta-analysis	Sadek Z, Salami A, Joumaa WH, Awada C, Ahmaidi S, Ramadan W. (2018)	France	Systematic review and meta-analysis	The objective of this study was to evaluate the effects of inspiratory muscle training on inspiratory muscle strength, functional capacity and dyspnoea for patients with chronic heart failure, by summarising the published research on the effects of inspiratory muscle	203 CHF patients In chronic heart failure patients,	Pimax, exercise duration, 100m walking test, QOL, Pimax, Pemax, IME, PFTs peak VO2, 12MWT, dyspnoea, SMIP, 6MWT, QOL, depression	Two IMT methods were analysed: inspiratory resistive training and threshold loading.	Typical training protocols involved training three, six or seven times per week with intensity ranging from 30% to 60% and for a duration ranging from 6 to 12 weeks. Maximal inspiratory pressure, walking distance and dyspnoea were improved in all studies and especially in those who set a load of 60% in their maximal inspiratory pressure, and have trained patients six times per week for 12 weeks. In chronic heart

					training. To identify the best mode of intervention in terms of: the load of maximal inspiratory pressure; the frequency of sessions; and the total duration of intervention				failure patients, inspiratory muscle training results in a marked improvement in inspiratorymuscle strength, walking distance and dyspnoea, notably when training patients at 60% of maximal inspiratory pressure, six times per week and for 12 weeks.
**1.a**	Meta-analysis of the effect of a pulmonary rehabilitation program on respiratory muscle strength in patients with chronic obstructive pulmonary disease	Eun Nam Lee, Moon Ja Kim (2018)	Republic of Korea	Systematic review. Meta-analysis	Pulmonary rehabilitatio n (PR) programs are important in the treatment of patients with chronic obstructive pulmonary disease (COPD) but vary widely in type, duration, and efficacy. This meta-analysis investigated the effect of PR programs	Patients with COPD	Maximal expiratory pressure (MEP) and maximal inspiratory pressure (MIP) and modified Borg score after the 6-min walking test (6MWT), percent predicted forced expiratory volume in 1 second (FEV1%pred), and FEV1/forced vital capacity (FVC) as a percentage	Respiratory muscle training (RT)	The PR programs had a significant effect on the MEP (SMD, 0.87; 95% CI, 0.42-1.32; p < .001), MIP (SMD, 0.53; 95% CI, 0.13-0.93; p = .009), and modified Borg score (SMD, -0.37; 95% CI, -0.52 to -0.22; p < .001) in patients with COPD. There was no effect on FEV1%pred (SMD, 0.09; 95% CI, -0.12 to 0.30; p = .406) or FEV1/FVC% (SMD, 0.04; 95% CI, -0.17 to 0.26; p = .702). PR programs improve respiratory muscle strength in patients

					on respiratory muscle strength in patients with COPD				with COPD. Strategies for selecting a suitable PR program need to be developed, and future studies should evaluate the long-term effects of such programs on pulmonary function.
**1.b**	Inspiratory Muscle Rehabilitation in Critically Ill Adults. A Systematic Review and Meta-Analysis.	Vorona S, Sabatini U, Al-Maqbali S, et al. (2018)	Canada	Systematic review and meta-analysis	The primary objective was to describe the range and tolerability of published methods for IMT. The secondary objectives were to determine whether IMT improves respiratory muscle strength and clinical outcomes in critically ill patients.	A total of 28 studies (n=1185) were included	maximal inspiratory pressure, MEP, clinical outcomes (duration of ventilation, duration of weaning, mortality, ICU and hospital length-of-stay, adverse events).	IMT was initiated during early mechanical ventilation, after patients proved difficult to wean (14 studies), or after extubatio. strength-training regimens, endurance-training regimens	IMT improved maximal inspiratory pressure compared to control (15 trials; mean increase 6 cm H2O, 95% CI 5-8 cm H2O; pooled relative ratio of means 1.19, 95% CI 1.14-1.25) and maximal expiratory pressure (4 trials, mean increase 9 cm, IMT was associated with a shorter duration of ventilation (9 trials, mean difference 4.1 days, 95% CI 0.8-7.4 days) and duration of weaning (8 trials, mean difference 2.3 days, [95% CI 0.7-4.0 days]) IMT in critically ill adults is

									feasible and well-tolerated and can achieve a modest but potentially meaningful improvement in respiratory muscle strength. The potential impact of IMT on clinical outcomes and long-term functional status and quality of life requires future confirmation. Based on presently available evidence, we suggest that IMT is feasible and safe in mechanically ventilated patients. Based on the foregoing considerations regarding tension-time index, clinicians may consider using IMT to improve diaphragm strength in patients who prove difficult to liberate from ventilation because of diaphragm weakness.

**1.b**	Results of Physiotherapy Treatments in Exacerbations of Chronic Obstructive Pulmonary Disease: A Systematic Review	Torres-Sánchez I, Cruz-Ramírez R, Cabrera-Martos I, Díaz-Pelegrina A, Valenza MC.(2017)	Spain	Systematic review	The objective of this study was to review clinical trials of the effectiveness of physiotherapy compared with standard care, focused mainly on the functional status of patients hospitalized for acute exacerbation of chronic obstructive pulmonary disease (AECOPD).	Adults with AECOPD. The number of patients included in all the studies was 1648.	Dyspnea, respiratory function, quality of life, and variables related to functional status	Chest physical therapy, breathing techniques, exercise, electrostimulation, and a combination of treatment modalities (Table 3)	The review revealed that for patients hospitalized for AECOPD, exercise, neuromuscular electrical stimulation, breathing exercises, and chest therapy significantly improved their functional status compared with standard care
**1.a**	Evidence regarding patient compliance with incentive spirometry interventions after cardiac, thoracic and abdominal surgeries: A	Narayanan AL, Hamid SR, Supriyanto E. (2016)	Malaysia	Systematic review	To explore the status of evidence on patient compliance with ISy interventions in randomized controlled trials (RCTs)	The search strategy yielded 527 records. 54 relevant RCTs retrieved for review, 18 excluded, 35 obtained. Of a total 3753 patients involved in these studies,	Session frequency<br>Inspiration frequency<br>Volume target<br>Breath hold (duration not specified)<br>Flow rate	ISy prescriptions: Six different ISy usage parameters were identified from these prescriptions: Session duration, Session frequency , Inspiration frequency ,	ISy parameters were identified in ISy prescriptions from these trials. There is a scarcity and inconsistency of evidence regarding ISy compliance. Compliance data should be obtained

	systematic literature review				in the above contexts	1957 (52.1%) had received ISy interventions.		Volume targets, Breath hold – the duration which the patient was to hold their breath at maximal inspiration. Flow rate – how quickly, or the speed at which each inspiration should be performed.	using reliable and standardized methods to facilitate comparisons between and among trials. These should be reported comprehensively to facilitate valid inferences regarding ISy intervention effectiveness.
**1.a**	Influence of inspiratory muscle training on weaning patients from mechanical ventilation: a systematic review	MSVolpe, AA Aleixo, PRM Negreiros de Almeida (2016)	Brazil	Systematic review	The aim of this systematic literature review was to evaluate the effectiveness of inspiratory muscle training in weaning patients from mechanical ventilation and to identify the most effective type of training for this	Adults (aged 18 years or older) receiving IMV in which pressure threshold devices. A total of 267 patients participated in the five randomized clinical trials analyzed here.	Maximal inspiratory pressure (PImax), ventilator weaning duration, success rate in weaning IMV, reintubation rate, and length of ICU and hospital stay	IMT	IMV duration before onset of training varied greatly among subjects. Three studies performed IMT using a threshold device and two studies used adjustments of ventilator pressure sensitivity. Four studies have shown that IMT resulted in a significant increase in inspiratory maximal pressure. Only two studies, however, have reported that IMT resulted in higher success rates in weaning patients from IMV. One study has found

					particular purpose.				that patients showed a shorter ventilator weaning duration after IMT.
**1.a**	Pre and postoperative inspiratory muscle training in patients undergoing cardiac surgery: Systematic review and meta-analysis	Gomes Neto M, Martinez BP, Reis HF, Carvalho VO. (2016)	Brazil	Systematic review and Meta-analysis	To determine the effects of pre- and postoperative inspiratory muscle training on length of postoperative hospital stay and pulmonary function in patients undergoing cardiac surgery	Cardiac surgery patients were included in this systematic review: Eight studies fulfilled the inclusion criteria. Four were about preoperative inspiratory muscle training (416 patients), three about postoperative inspiratory muscle training (115 patients) and one study about pre- and postoperative inspiratory muscle training (43 patients).	HR, SBP, DBP, RPE, PPC and lung function; MIP and muscle endurance, PPC and length hospitalization; Spirometry, arterial blood gases, PPC, MIP and muscle endurance; Spirometry, arterial blood gases, PPC, MIP, MEP; MIP, MEP, PEF, pain, hospitalization days and TV; MIP, MEP, VC, TV; MIP, MVV, Pulmonary function testing, 6MWT, quality of life	Interventions include early mobilization, breathing exercises, coughing techniques, incentive spirometry, continuous positive airway pressure and respiratory muscle training.	Preoperative inspiratory muscle training resulted in improvement in: Reduction in length of postoperative hospital stay of −2days (95% CI −3.4, −0.7, N=302), inspiratory pressure of 16.7 cmH2O (95% CI 13.8, 19.5, N=386), forced expiratory volume in one second of 3% predicted (95% CI 0.1, 6, N=140), forced vital capacity of 4.6% predicted (95% CI 1.9, 7.4, N=140). Patients that received preoperative training had an inspiratory muscle training reduced risk of postoperative

									pulmonary complications, (RR=0.6; 95% CI 0.5 to 0.8; P=0.0004, N=386). Postoperative inspiratory muscle training resulted in improvement in inspiratory pressure of 16.5 cmH2O (95% CI 4.9, 27.8, N=115), and tidal volume of 185ml (95% CI 19.7, 349.8, N=85).Pre- and postoperative inspiratory muscle training showed to be a beneficial intervention in the treatment of patients undergoing cardiac surgery, especially when performed in the preoperative period and associated pre- and postoperative. Besides the effects of reduced hospitalization, there was improvement in lung function, which justifies its use for the prevention of postoperative

									complications in cardiac surgery patients.

**1.b**	Pulmonary physical therapy techniques to enhance survival in amyotrophic lateral sclerosis: a systematic review	Macpherson CE, Bassile CC (2016)	USA	Systematic review	The purpose of this systematic review was to examine the effectiveness of pulmonary physical therapy interventions across the progressive stages of ALS.	Participants diagnosed with ALS according to the revised El Escorial criteria	Outcome measures included but not limited to standardized measures of respiratory function (eg, FVC, forced expiratory volume in the first second of expiration, total lung capacity, vital capacity, and PCEF).	Nonmechanical pulmonary interventions, including Diaphragmatic breathing, IMT, Lung Volume Recruitment Training , and MAC techniques	With the exception of diaphragmatic breathing, pulmonary physical therapy interventions were effective in improving multiple respiratory outcome measures in this population. Inspiratory muscle training (IMT) was shown to prolong respiratory muscle strength with a strong effect size (ES = 1.48) for FVC. In addition, mean length of survival increased by 12 months. Lung volume recruitment training (LVRT) strongly enhanced immediate cough efficacy with improved FVC (ES = 1.02) and PCEF (ES = 1.82). Manually assisted cough (MAC) only improved PCEF by a small amount (ES = 0.15, bulbar ALS; ES = 0.16, classical ALS groups). interventions

									including IMT, LVRT, and MAC have at least moderate strength for effectiveness in improving respiratory outcome measures, participation level, and increasing survival.
**1.b**	Effect of respiratory rehabilitation techniques on the autonomic function in patients with chronic obstructive pulmonary disease: A systematic review.	Mohammed J, Da Silva H, Van Oosterwijck J, Calders P. (2016)	Belgium	Systematic review	The aim of this systematic review is to provide a grade 2 evidence to support the effects of three distinct respiratory rehabilitation techniques, namely, con trolled breathing, NIMV application, and oxygen sup plementatio	A total of 322 (197 males) patients with COPD	herat Rate variability, BRS baroreceptor sensitivity	controlled breathing techniques, noninvasive mechanical ventilation (NIMV), and oxygen supplementation	This review has showed that a variety of respira tory rehabilitation techniques may have a beneficial influence that can be clinically applied for the man agement of impairments of the sympathetic and para sympathetic systems, hence making important modifications in the cardiovascular health possible among patients with COPD. Finally, the findings from our systematic review will help to

					n on the AF parameters in patients with COPD.				draw the attention of researchers in the field of rehabilitation of chronic respiratory disease to the impact of conservative treat ment approaches on the extrapulmonary system.
**1.a**	Inspiratory muscle training facilitates weaning from mechanical ventilation among patients in the intensive care unit: a systematic review	Elkins M, Dentice R. (2015)	Australia	Systematic review	Does inspiratory muscle training improve inspiratory muscle strength in adults receiving mechanical ventilation? Does it improve the duration or success of weaning? Does it affect length of stay, reintubation, tracheostom y, survival, or the need	Adults receiving mechanical ventilation - age > 16, intubated or tracheostomised, receiving mechanical ventilation in ICU. 394 participants	Inspiratory muscle strength, rapid shallow breathing index, weaning duration, weaning success, duration of mechanical ventilation, reintubation, tracheostomy, length of stay, non-invasive ventilation, survival, tolerability, adverse effects	Inspiratory muscle training via any of the following: isocapnic/normoc apnic hyperpnoea, inspiratory resistive training, threshold pressure training, adjustment of ventilatior pressure trigger sensitivity	Random-effects meta-analyses showed that the training significantly improved maximal inspiratory pressure (MD 7 cmH2O, 95% CI 5 to 9), the rapid shallow breathing index (MD 15 breaths/min/l, 95% CI 8 to 23) and weaning success (RR 1.34, 95% CI 1.02 to 1.76). Although only assessed in individual studies, significant benefits were also reported for the time spent on non-invasive ventilation after

					for post-extubation non-invasive ventilation? Is it tolerable and does it cause adverse events?				weaning (MD 16 hours, 95% CI 13 to 18), length of stay in the intensive care unit (MD 4.5 days, 95% CI 3.6 to 5.4) and length of stay in hospital (MD 4.4 days, 95% CI 3.4 to 5.5). Weaning duration decreased in the subgroup of patients with known weaning difficulty. The other outcomes weren’t significantly affected or weren’t measured. Inspiratory muscle training for selected patients in the intensive care unit facilitates weaning, with potential reductions in length of stay and the duration of non-invasive ventilatory support after extubation.

**1.b**	Systematic Review of Inspiratory Muscle Training After Cerebrovascular Accident	Martín-Valero R, De La Casa Almeida M, Casuso-Holgado MJ, Heredia-Madrazo A.. (2015).	Spain	Systematic review and meta-analysis	The main aim of this review was to detect the level of evidence and grade of recommendation according to the inspiratory muscle training interventions of subjects after stroke.	First, subjects who were included had experienced a moderate or severe stroke according to the National Institutes of Health Stroke Scale with a score of 5–25 at the time of admission	the outcome measures included functional max imum inspiratory pressure (PImax),8 maximum expiratory pressure (PEmax), respiratory muscle strength, FVC,5 in spiratory capacity, and forced expiratory peak	the intervention included inspiratory exercises of different intensities and duration through resistance, which was controlled by a threshold valve adapted to the needs and changes of peo ple after stroke as soon as subjects progressed through the study.	This research has examined levels of evidence and rec ommendation grades of various therapeutic interventions of inspiratory muscle training in people who have suffered a stroke. It has been observed that respiratory muscle train ing can improve strength and endurance of respiratory muscles in these subjects. At least one 8-week intervention carried out at a frequency of 3 to 6 days/week is recom mended. Each session should last between 15 and 30 min utes per set with one or 2 daily sessions per day at an intensity of 30–40% of maximal inspiratory pressure. It is necessary to use the suitable spirometer for each person. The results of this research support

									physiotherapy respi ratory techniques in people who have suffered a stroke.
**1.a**	Postoperative outcomes following preoperative inspiratory muscle training in patients undergoing cardiothoracic or upper abdominal surgery: a systematic review and meta analysis	Mans, C. M., Reeve, J. C., & Elkins, M. R. (2015)	New Zealand	A systematic review and meta analysis of randomized controlled trials (or quasirandom ized controlled trials)	To evaluate whether preoperative inspiratory muscle training is effective in preventing postoperativ e pulmonary complication s and reducing length of hospital stay in people undergoing cardiothorac ic or upper abdominal surgery.	Eight studies involving 295 participants. Participants were adults (16years and over) awaiting elective open cardiac, thoracic, or upper abdominal surgery.	respiratory muscle strength, inspiratory muscle endurance, exercise tolerance, pulmonary function, duration of postoperative ventilation, oxygenation,	Inspiratory muscle training	Preoperative inspiratory muscle training significantly improves respiratory (muscle) function. The trained group had significantly higher maximal inspiratory pressure at the end of the preoperative training period (mean difference: 15cmH2O, 95% confidence interval (CI): 9 to 21). This benefit was maintained through the early postoperative period, when lung

									function also recovered significantly more quickly in the trained group. Inspiratory muscle training also substantially reduced postoperative pulmonary complications (relative risk 0.48, 95% CI 0.26 to 0.89). Although not statistically significant, length of hospital stay also tended to favour the trained group. TParticipant satisfaction with inspiratory muscle training was high. in the early postoperative period, halving the risk of pulmonary complications.
**1.a**	Influence of Inspiratory Muscle Weakness on Inspiratory Muscle Training Responses in Chronic Heart	Montemezzo D, Fregonezi GA, Pereira DA, Britto RR, Reid WD. (2014)	Canada	Systematic review and Meta-analysis	To determine whether the impact of inspiratory muscle weakness on inspiratory muscle	Participants with CHF and left ventricular ejection fraction decreased, >18 years old	6-minute walk distance, maximal inspiratory pressure, sustained maximal inspiratory pressure,	IMT	The existing data suggest that IMT is an effective intervention for improving exercise capacity, mainly in patients with CHF with inspiratory muscle weakness.

	Failure Patients: A Systematic Review and Meta-Analysis				training (IMT) affects inspiratory function and exercise capacity in chronic heart failure (CHF) patients		inspiratory muscle weakness, peak oxygen consumption, minute ventilation,		The most significant novel finding of this systematic review is that people with CHF who had weaker inspiratory muscles showed greater improvements inmaximal and submaximal exercise capacities after IMT.
**1.a**	Expiratory and Expiratory Plus Inspiratory Muscle Training Improves Respiratory Muscle Strength in Subjects with COPD: Systematic Review	Neves LF, Reis MH, Plentz RD, Matte DL, Coronel CC, Sbruzzi G. (2014)	Brazil	Systematic review and Meta-analysis	The aim of this study was to systematically review the effects of EMT and EMT plus IMT compared to control groups of COPD subjects	EMT versus a control group or EMT plus IMT versus a control group in subjects with COPD,	Maximum expiratory pressure (PEmax, cm H2O), maximum inspiratory pressure (PImax, cm H2O), distance (or exercise tolerance) in 6MWT (meters), and dyspnea (Borg scale)	EMT, EMT combined with IMT	We observed that EMT provided higher gain in maximum expiratory pressure (PEmax 21.49 cm H2O, 95% CI 13.39 – 29.59) and maximum inspiratory pressure (PImax 7.68 cm H2O, 95% CI 0.90 – 14.45) compared to control groups. There was no significant difference in the 6-min walk test distance (29.01 m, 95% CI 39.62 to 97.65) and dyspnea (0.15, 95% CI 0.77 to 1.08). In relation to EMT plus IMT, we

									observed that PEmax (31.98 cm H2O, 95% CI 26.93–37.03) and PImax (27.98 cm H2O, 95% CI 20.10 –35.85) presented higher values compared to control groups.In thismeta-analysis, we found that EMT is an effective way to improve strength of the inspiratory and expiratory muscles. Hence, as expected, the combination ofEMTandIMTshowed very good results for improving respiratory muscle strength.
**1.a**	Preoperative intervention reduces postoperative pulmonary complications but not length of stay in cardiac surgical patients: a systematic review	Snowdon D, Haines TP, Skinner EH. (2014)	Australia	Systematic review with meta-analysis of (quasi) randomised trials	Does preoperative intervention in people undergoing cardiac surgery reduce pulmonary complications, shorten length of stay in the intensive care unit	People undergoing coronary artery bypass grafts and/or valvular surgery, n=2689, mainly males	Time to extubation, length of stay in ICU and hospital (reported in days), postoperative pulmonary complications, physical function	Any intervention, such as education, inspiratory muscle training, exercise training or relaxation, delivered prior to surgery to prevent/reduce postoperative pulmonary complications or to hasten recovery of	Preoperative intervention significantly reduced the time to extubation (MD -0.14 days, 95% CI -0.26 to -0.01) and the relative risk of developing postoperative pulmonary complications (RR 0.39, 95% CI 0.23 to 0.66).<br>However, it did not

					(ICU) or hospital, or improve physical function?			function (interventions given by nurse, kinesiologist, exercise specialist, physiotherapist, physician, ocupational therapist)	significantly affect the length of stay in ICU (MD -0.15 days, 95% CI -0.37 to 0.08) or hospital (MD -0.55 days, 95% CI -1.32 to 0.23), except among older participants (MD -1.32 days, 95% CI -2.36 to -0.28). When the preoperative interventions were separately analysed, inspiratory muscle training significantly reduced postoperative pulmonary complications and the length of stay in hospital.
**1.b**	The effects of high intensity exercise during pulmonary rehabilitation on ventilatory parameters in people with moderate to severe stable COPD: a systematic review	Osterling K, MacFadyen K, Gilbert R, Dechman G. (2014)	Canada	Systematic review	The objective of this systematic review was to determine whether people with moderate to severe COPD who are participating in pulmonary rehabilitatio	Males and females with moderate to severe, stable COPD were included in this study. Participant numbers ranged from 18 to 48 people per study, and a pooled total of 125 participants. The age range of the participants	Minute ventilation measured in liters per minute, respiratory rate measured in breaths per minute, and tidal volume measured in liters. graded exercise testing that measured tidal volume,	Pulmorary rehabilitation - All programs delivered pulmonary rehabilitation on an outpatient basis. All programs included aerobic training on treadmills or cycle ergometers. Two of the programs also	Participants in three studies trained at high intensity (70%–80% maximum workload), demonstrating statistically significant changes in tidal volume and respiratory rate. One study did not demonstrate positive ventilatory benefits; however, participants may not

					n and exercising at high intensity demonstrate the changes in ventilatory parameters that are associated with decreased dyspnea.	was from 40 to 80 years, and males represented 63% of the total review population.	respiratory rate, minute ventilation, and inspiratory capacity	included resistance training. The duration of the pulmonary rehabilitation interventions ranged from 4 to 12 weeks, 3–5 times each week, with sessions lasting 90–180 minutes. breathing techniques, nutrition, and energy conservation	have met the desired training intensity. Two studies reported improvement in dyspnea at submaximal exercise intensities. One study noted an increased maximum workload with no significant change in dyspnea at peak exercise. People with moderate to severe, stable COPD were able to perform high intensity exercise, which was associated with positive changes in ventilatory parameters and dyspnea.

**1.b**	Pulmonary Rehabilitation for Mild COPD: A Systematic Review	Jácome,C ; Marques, A. (2014)	Portugal	Systematic review	Assess the impact of pulmonary rehabilitation on exercise capacity, HRQOL, health-care resource use, and lung function in patients with mild COPD	Patients with mild COPD (FEV1 » 80% predicted). Total of 100 participants (1 - no data on age/gender ratio, n=31; 2 and 3: age ranged from 41 to 83, 47 male:22 female)	Exercise capacity (6MWD), HRQOL, Health-care resourse use, lung function	out-patient programs, with duration between 6 and 8 weeks and frequency between 2 and 3 sessions a week. The exercise training sessions lasted between 60 and 90 min, and included mainly aerobic training, strength training, and respiratory muscle training. Both programs included an educational component. Another, implemented a home-based pulmonary rehabilitation program, consisting of 1 week of pursed-lip breathing and aerobic training under the supervision of health professionals followed by 6 months of peer-led walking and participation in	Significant improvements in exercise capacity (effect size [ES] 0.87–1.82) and HRQOL (ES 0.24 – 0.86) were found when comparing pretest-posttest data and when comparing PR with standard medical treatment. In one study, a significant decrease in hospitalization days was found (ES 0.38). No significant effects were observed on the number of emergency department visits (ES 0.32), number of hospitalizations (ES 0.219), or lung function (ES 0.198).

								ball games for 60 min twice a week.	
**1.b**	Training of Respiratory Muscles in Patients With Multiple Sclerosis: A Systematic Review	Martín-Valero, R., Zamora-Pascual, N., & Armenta-Peinado, J. A. (2014)	Spain	Systematic review	The aim of this systematic review was to summarize the level of evidence and grades of recommenda tion regarding therapeutic respiratory muscle training intervention s in pa tients with multiple	The population included samples of people independent in activities of daily living and wheelchair or bedridden patients with MS	PImax, maximum expiratory pressure [PEmax], re spiratory muscle strength), physical capacity (6-min walk test [6MWT]),clinica l outcomes (severity of the dis ease, Kurtzke Expanded Disability Status Scale [EDSS]	the intervention included inspiratory and expiratory exercises of different intensities and duration through a resistance offered by a value threshold, adapting to the needs and changes of the individual while progressing through the study.	Respiratory muscle training may improve respiratory muscle function in patients with MS. However, the paucity of studies in the area and the variability between them are limiting factors. According to the studies included in this systematic re view, the training protocols in which the expi rations are done in a specific way achieve greater results in PEmax, and trials that focus

					sclerosis (MS)				on training respiratory muscles through inspirations obtain greater results in PImax. Both types of training (strength and endurance) show a real improvement in muscle endurance, but only strength train ing is able to significantly improve PImax, PEmax, and func tional exercise capacity.
**1.b**	A systematic review of pre-surgical exercise intervention studies with cancer patients	Singh F, Newton RU, Galvão DA, Spry N, Baker MK. (2013)	Australia	Systematic review	Systematically review the available literature regarding pre-surgery exercise training interventions in cancer patients and examine their effects on physiological outcomes as well as quality of life (QOL) and length	The studies included participants diagnosed with one of three different types of cancer. 11 - lung cancer patients, 4 - prostate cancer and the rest related to cancers of the abdominal area (colon, colorectal, liver, etc). The 18 articles consisted of 966 participants (between 54.1 and 71.1)	Data on physiological and functional performances as well as QOL indices and length of hospital stay	exercise intervention and Breathing exercises	Mode, frequency, duration, and intensity of exercise intervention varied across the different cancer groups. The majority of studies showed preliminary positive change in clinical outcomes with significant improvements in the rate of incontinence, functionalwalking capacity and cardiorespiratory fitness. Pre-surgical exercise may benefit cancer patients

					of hospital stay				through positive effects on function and physical capacity. Surgical oncologists may consider pre-surgical exercise interventions as a potential adjuvant therapy to improve patients' outcomes.
**1.b**	Preoperative aerobic exercise training in elective intra-cavity surgery: a systematic review	O'Doherty AF, West M, Jack S, Grocott MP. (2013)	UK	Systematic review	Check if preoperative aerobic exercise training in intra-cavity surgery result in improved postoperative clinical outcomes. Secondary objectives were to describe the effect of such an intervention on physical fitness and health-related quality of life (HRQL) and report feasibility,	Studies recruiting human adult participants awaiting major cardiac, respiratory, or gastrointestinal surgery were included in this review	Peak oxygen uptake (V˙O2 peak), the highest oxygen uptake measured during a symptom limited maximal exercise test; the anaerobic threshold (AT), the oxygen consumption at which muscle energy synthesis during exercise is no longer wholly fuelled by aerobic metabolism; and 6 min walk distance (6MWD), the maximum distance walked in 6 min	1- aerobic interval; 2- aerobic continuous; 3- aerobic continuous, strength, breathing exercises; 4- aerobic continuous, strength; 5- high intensity continuous, breathing and abdominal muscke exercises; 6- aerobic continuous, strength; 7- aerobic continuous, strength, breathing exercises; 8- aerobic continuous and	One study in cardiac surgery demonstrated reduced postoperative hospital and intensive care length of stay in the intervention group. Eight studies showed improvement in ≥1 measure of physical fitness after the intervention. HRQL was reported in five studies; three showed improved HRQL after the intervention. The frequency, duration, and intensities of the exercise interventions varied across the studies. Adherence to exercise

					safety, and cost-effectiveness			interval; 9-aerobic continuous; 10-aerobic continuous	interventions was good. Two exercise-related adverse events (transient hypotension) were reported. Evidence for improved postoperative clinical outcome after preoperative aerobic exercise training interventions is limited. However, preoperative aerobic exercise training seems to be generally effective in improving physical fitness in patients awaiting intra-cavity surgery and appears to be feasible and safe.
**1.a**	Inspiratory muscle training for asthma (review)	Silva IS, Fregonezi GA, Dias FA, Ribeiro CT, Guerra RO, Ferreira GM. (2013)	Brazil	Systematic review of RCT's	To evaluate the efficacy of inspiratory muscle training with either an external resistive device or threshold loading in people with asthma.	We included five studies involving 113 adults. Participants in four studies had mild to moderate asthma and the fiJh study included participants independent of their asthma severity.	Primary outcomes (inspiratory muscle strength, exacerbations requiring a course of oral or inhaled corticosteroids or emergency department visits), secondary outcomes	IMT (normocapnic hyperpnoea, flow resistive loading and pressure threshold loading)	The studies showed a significant improvement in inspiratory muscle strength (PImax). Results from one study showed no significant diEerence between the training group and the control group (no treatment or usual care) for expiratory muscle

							(inspiratory muscle endurance, expiratory muscle strength, lung function, asthma symptoms - measures of dyspnoea or breathlessness with Borg score or a Visual Analogue Scale (VAS), hospital admissions, use of reliever medication, days off school)		strength, lung function, sensation of dyspnoea (breathlessness) and use of reliever medication. Given the insuEicient evidence found in this review, we believe that there is a need for more well conducted studies in order to assess the eEicacy of IMT in people with asthma, including children.
**1.a**	Efficacy of inspiratory muscle training in chronic heart failure patients: A systematic review and meta-analysis	Smart NA, Giallauria F, Dieberg G. (2013)	Australia, Italy	Systematic review and Meta-analysis	Determine magnitude of change in peak VO2, six minute walk distance (6MWD), Quality of Life measured by the Minnesota Living with Heart Failure Questionnair e (MLWHFQ),	CHF patients undergoing IMT, for a minimum of 2 weeks, were included in this study. The eleven included studies contained data on 287 participants: 148 IMT participants and 139 sham or sedentary control.	The primary outcome measure following intervention was post exercise change in peak VO2, in ml kg− min−1. Secondary outcomes were changes in 6MWD, MLHFQ, PImax, and VE/VCO2 slope	IMT	Compared to control groups, CHF patients undergoing IMT showed a significant improvement in peak VO2 (+1.83 ml kg−1 min−1, 95% C.I. 1.33 to 2.32 ml kg−1 min−1, pb0.00001); 6MWD (34.35 m, 95% C.I. 22.45 to 46.24 m, pb0.00001); MLWHFQ (−12.25, 95% C.I. −17.08 to −7.43, pb0.00001); PImax (+20.01, 95% C.I. 13.96 to 26.06,

					maximal inspiratory pressure (PI max) and ventilatory equivalent for carbon dioxide (VE/VCO2 slope) with IMT.				pb0.00001); and VE/VCO2 slope (−2.28, 95% C.I. −3.25 to −1.30, pb0.00001).
**1.a**	Effect of diaphragmatic breathing exercise on quality of life in subjects with asthma: A systematic review	Prem V, Sahoo RC, Adhikari P. (2013)	India	Systematic review, RCT	The aim of this systematic review was to determine if diaphragmatic breathing exercise improves quality of life (QoL) in asthma	Asthma patients vs control (mean age between 45 and 48): n=40 (20 asthma)/ n=183 (94 asthma)/ n=31 (17 asthma)	ACR, SF-36, NQ, ETCO2, FEV1% // AQLQ score, with secondary outcomes including spirometry, bronchial hyperresponsiveness, exhaled nitric oxide, induced sputum eosinophil count and Asthma Control Questionnaire, hospital anxiety and depression, and hyperventilation (Nijmegen) // AQLQ and Nijmegen scores	Breathing retraining (20 min each day, 2-3 times/day, 6 months) // Diaphragmatic breathing (10 minutes each day, 6 months) // Diaphragmatic breathing (10 minutes each day, 6 months)	Breathing training lead to significant improvement in ACT, ETCO2, reduced respiratory rate, increased FEV1%<br>Breathing training resulted in improvements in asthma-specific health status and other patient-centered measures but not in asthma pathophysiology. Improved outcomes in the overall score, symptoms, and environment domains were seen. The results demonstrate that breathing exercise improves QoL in the short term and long term compared to

									asthma medication and education. There is evidence for mechanism on effect of breathing retraining on asthma through reduction of hyperventilation measured with increased ETCO2 and decreased respiratory rate.
**1.a**	Breathing exercises in upper abdominal surgery: a systematic review and meta-analysis.	Grams ST, Ono LM, Noronha MA, Schivinski CI, Paulin E. (2012)	Brazil	systematic review and meta-analysis	To undertake a systematic review of randomized and quasi-randomized studies that assessed the effects of breathing exercises on the recovery of pulmonary function and prevention of PCCs after upper abdominal surgery UAS.	pre- and postoperative UAS patients, in which the primary intervention was breathing exercises without the use of incentive inspirometers (N=132)	Data on maximal respiratory pressures MIP and MEP, spirometry, diaphragm mobility, and postoperative complications were extracted and analyzed.	breathing exercises without the use of incentive inspirometers	the breathing exercises were likely to have induced MEP and MIP improvement treatment effects of 11.44 mmH2O (95%CI 0.88 to 22) and 11.78 mmH2O (95%CI 2.47 to 21.09), respectively. Breathing exercises are likely to have a beneficial effect on respiratory muscle strength in patients submitted to UAS. The present review found a significant improvement for maximal respiratory pressures in patients who performed breathing exercises.

									Interestingly, these findings are related to breathing exercises without resistance commonly used in muscle training, therefore the increase in respiratory pres sures may be related to the characteristics of the exercises. In the studies that found improved MIP and MEP in the groups that performed breathing exercises, the programs consisted of diaphragm breathing, sustained maximal inspiration, and fractional inspiration aimed at increasing diaphragm mobil ity, improving respiratory muscle synergism, and maintaining muscle trophism by using the diaphragm and reducing the ac tion of accessory muscles

**1.a**	The impact of home-based physiotherapy interventions on breathlessness during activities of daily living in severe COPD: A systematic review	Thomas MJ, Simpson J, Riley R, Grant E.(2010)	UK	Systematic review	To conduct a systematic review and meta-analysis to determine the impact of home-based physiotherapy interventions on breathlessness during activities of daily living (ADL) in severe chronic obstructive disease (COPD).	Individuals over 18 years of age with severe COPD (defined as forced expiratory volume in 1 second ≤50% predicted) without cardiovascular co-morbidities	1- CRDQ, 2- CRDQ, 3- CRDQ, 4- BDI/TDI, 5- CRDQ + BDI/TDI, 6- BDI/TDI, 7- BDI/TDI	expiratory muscle training. inspiratory muscle training	Statistically significant breathlessness ADL outcome improvements were reported for all interventions except expiratory muscle training. Five studies demonstrated clinical significance (four for inspiratory muscle training and one for exercise). The random-effects meta-analysis indicated that, on average, inspiratory muscle training improved the breathlessness score significantly by 2.36 (95% confidence interval 0.76 to 3.96) compared with controls.

**1.a**	Short-term effects on outcomes related to the mechanism of intervention and physiological outcomes but insufficient evidence of clinical benefits for breathing control: a systematic review	Lewis LK, Williams MT, Olds T. (2007)	Australia	Systematic review with meta-analysis	What is the volume, quality, consistency, and generalisability of the evidence for breathing control? What is the effect on outcomes related to the target and mechanism of breathing control, as well as physiological and clinical outcomes?	People with chronic respiratory disease, post-surgical, or asymptomatic individuals.	Abdominal excursion, diaphragm excursion, respiratory rate, tidal volume, ventilation, vital capacity, forced vital capacity (FVC), expiratory flow rate, forced expiratory volume in one second (FEV1), respiratory muscle strength, oxygen consumption (VO2), work of breathing, respiratory muscle efficiency, 12-minure walk test, ventilation distribution (regional clearance), arterial oxygen saturation (SaO2), percutaneous gases, dyspnoea	Breathing control (relaxed basal, diaphragmatic, or abdominal breathing)	Twenty studies were included within the meta-analysis. A beneficial effect was found for abdominal movement (SMD 1.36, 95% CI 0.42 to 2.31), diaphragm excursion (SMD 1.39, 95% CI 1.00 to 1.77), respiratory rate (SMD –0.84, 95% CI –1.09 to –0.60), tidal volume (SMD 0.98, 95% CI 0.71 to 1.25), arterial oxygen saturation (SMD 0.63, 95% CI 0.25 to 1.02) and percutaneous oxygen (SMD 1.48, 95% CI 0.85 to 2.11). Breathing control had a detrimental effect on the work of breathing (SMD 1.06, 95% CI 0.52 to 1.60) and dyspnoea (SMD 1.47, 95% CI 0.88 to 2.05)There was no clear evidence of an effect on ventilation, longer-term mechanisms such as

									pulmonary volume or flow, and respiratory muscle strength. Additionally, there was no overall effect on physiological outcomes related to the energy cost of breathing such as oxygen consumption and respiratory muscle efficiency, or outcomes related to gas exchange such as the distribution of ventilation.
**1.a**	Inspiratory muscle training compared with other rehabilitation interventions in adults with Chronic Obstructive Pulmonary Disease: A systematic literature review and meta-analysis	Crowe J, Reid WD, Geddes EL, O'Brien K, Brooks D. (2005)	Canada	Systematic review and meta-analysis	Determine the effect of inspiratory muscle training (IMT) (alone or combined with exercise and/or pulmonary rehabilitatio n) compared to other rehabilitatio n intervention s such as:	Patients with stable COPD. Thirteen of the 16 included studies enrolled more males than females. One included only males (26) and 1 included predominantly females (36). The mean age for subjects in most studies was between 56 and 72, however 3	Inspiratory muscle strength and endurance, exs capacity, dyspnea, PFT, quality of life, fatigue, pulmonary function	IMT with a general exercise program (lower extremity aerobic exercise, upper extremity training); IMT compared with education; IMT compared to other breathing techniques (intermittent positive pressure breathing, incentive spirometry	Results showed significant improvements in inspiratory muscle strength and endurance, and in the dyspnea scale on a quality of life measure, for participants in the IMT versus education group. IMT results in improved inspiratory muscle strength and endurance

					exercise, education, other breathing techniques or exercise and/or pulmonary rehabilitation among adults with chronic obstructive pulmonary disease (COPD).	included some subjects under the age of 50 years. The FEV1 was described as being less than 65% of predicted or less than 1.3 liters.		postural drainage and active cycles of breathing techniques (ACBT), conventional breathing re-training, or respiratory muscle stretch gymnastics; IMT combined with exercise/pulmonary rehabilitation (PR) compared to exercise/pulmonary rehabilitation (PR) alone or with sham	compared to education. Further trials are required to investigate the effect of IMT (or combined IMT) compared to other rehabilitation inventions for outcomes such as dyspnea, exercise tolerance, and quality of life.
